# Double-scattering/reflection in a Single Nanoparticle for Intensified Ultrasound Imaging

**DOI:** 10.1038/srep08766

**Published:** 2015-03-05

**Authors:** Kun Zhang, Hangrong Chen, Xiasheng Guo, Dong Zhang, Yuanyi Zheng, Hairong Zheng, Jianlin Shi

**Affiliations:** 1 State Key Laboratory of High Performance Ceramics and Superfine Microstructures, Shanghai Institute of Ceramics, Chinese Academy of Sciences, Shanghai 200050, P. R. China; 2 Key Laboratory of Modern Acoustics, MOE, Institute of Acoustics, Department of Physics, Nanjing University, Nanjing 210093, P. R. China; 3 Second Affiliated Hospital of Chongqing Medical University, Chongqing 400010, P. R. China; 4 Shenzhen Institutes of Advanced Technology, Chinese Academy of Sciences, Shenzhen 518055, P. R. China

## Abstract

Ultrasound contrast agents (UCAs) designed by the conventional composition-based strategy, often suffer from relatively low ultrasound utilization efficiency. In this report, a structure-based design concept of double-scattering/reflection in a single nanoparticle for enhancing ultrasound imaging has been proposed. To exemplify this concept, a rattle-type mesoporous silica nanostructure (MSN) with two contributing interfaces has been employed as the ideal model. Contributed by double-scattering/reflection interfaces, the rattle-type MSN, as expected, performs much better in *in vitro* and *in vivo* ultrasound imaging than the other two nanostructures (solid and hollow) containing only one scattering/reflection interface. More convincingly, related acoustic measurements and simulation calculations also confirm this design concept. Noticeably, the rattle-type MSN has also been demonstrated capable of improving intracellular ultrasound molecular imaging. As a universal method, the structure-design concept can extend to guide the design of new generation UCAs with many other compositions and similar structures (*e.g.*, heterogeneous rattle-type, double-shelled).

With increasingly prevalent applications of ultrasound equipments, ultrasound contrast agents (UCAs) have been becoming the research hotspot since the emergence of the concept of improving ultrasound imaging contrast by using UCAs in 1968^1^,^2^, To date, UCAs have experienced several generations of development^3^,^4^, However, the reported synthetic strategy for UCAs inevitably followed the usual way of the material composition design, including the composition design for outer shell and/or inner core medium. For example, inner core medium has been progressively developed from free gas bubbles, to gas bubbles encapsulated within outer shell, then to the liquid droplets encapsulated within outer shells^5^,^6^,^7^,^8^, while the composition of outer shell has been gradually upgraded from micelles^6^, polymers^9^, liposomes and proteins^8^,^10^, and to those inorganic carriers like silica^11^,^12^,^13^,^14^,^15^, However, no matter what compositions of inner core or outer shell were employed in all previously reported UCAs, they all share the same structure with only one contributing interface (*i.e.* the outer surface), and thus they can only realize once scattering/reflection in ultrasound imaging from the structural viewpoint, leading to the limited utilization of ultrasound waves^16^,^17^,^18^. Therefore, designing UCAs from the perspective of structure innovation that produces multi-scattering/reflection to greatly enhance the ultrasound (US) utilization efficiency is of great significance but still remains a great challenge.

Herein, from the structure design-point of view, we proposed a brand-new structure design-based concept of double-scattering/reflection in a single particle for the first time, which is completely different from conventional composition-based design strategy. Rattle-type mesoporous silica nanostructure (MSN) with two contributing interfaces that has been well documented in drug delivery^19^,^20^, was chosen as the ideal model to demonstrate this design concept *via* imaging outcome evaluations, simulation calculations and acoustic measurements, since rattle-type MSN can perfectly cater to the model requirements of structure-based design concept. Moreover, the influences of the second scattering cross-section in rattle-type MSN on ultrasound imaging performance have been also investigated, and the universality of such a structure design concept has been well demonstrated *via* designing and comparing UCAs of different structures or different compositions. As a general design/synthesis strategy, besides silica-based UCAs, this structural-based design strategy can be applied to guide the design of other compositions-based UCAs, *e.g.*, organic, hybrid or other types of inorganic-based UCAs, and this strategy is hopeful for overcoming the bottleneck problem of low ultrasound utilization efficiency (for the UCAs with the same composition but different structures, the ratio of single scattering to incident ultrasound is lower than that of twice or more scattering to incident ultrasound in a single nanoparticle), and promises a widespread application in designing new-generation UCAs.

## Results

### Model construction and imaging evaluations

**Figure 1:**
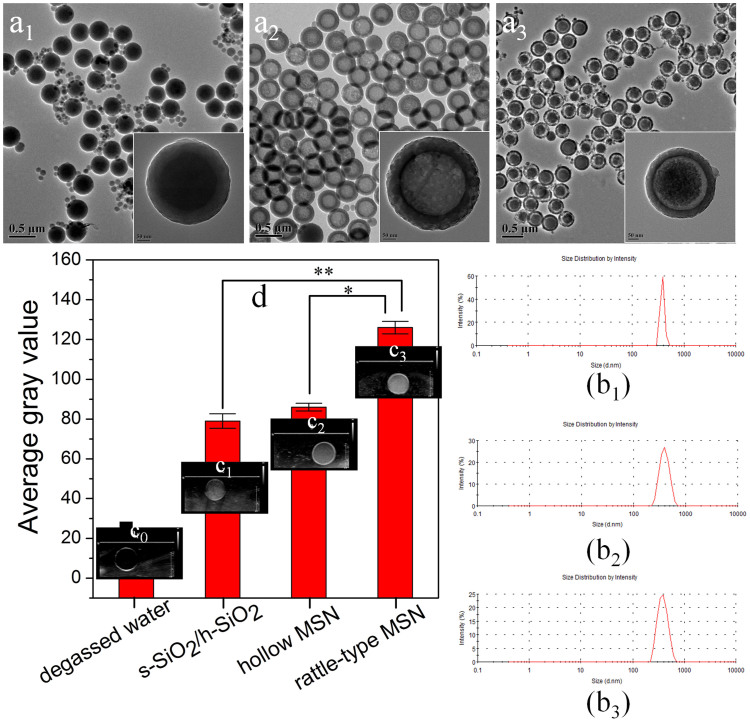
Structure characterization and Imaging capability evaluations. (a_1_–a_3_) TEM images of s-SiO_2_/h-SiO_2_, hollow MSN and rattle-type MSN, respectively; (b_1_–b_3_) Integral particle size distributions of s-SiO_2_/h-SiO_2_, rattle-type MSN and hollow MSN, respectively, *via* DLS; (d) Measured average gray values employing PBS, s-SiO_2_/h-SiO_2_, rattle-type MSN and hollow MSN as UCAs, and the insets (c_1_–c_4_) are their corresponding ultrasound images under B fundamental imaging mode, respectively. Notes: * and ** represent P ≤ 0.05 and P ≤ 0.01, respectively.

All the samples, *e.g.* rattle-type MSN, solid s-SiO_2_/h-SiO_2_, and hollow MSN, were respectively obtained *via* a well-developed method^21^. According to transmission electron microscopy (TEM) images and dynamic light scattering (DLS) data (Fig. 1a_1_–a_3_ and 1b_4_–b_3_), the average particle diameters of the three nanostructures with well-defined spherical morphology, high dispersity and narrow diameter distribution are 420 ± 30 nm, the thickness of outer shell is around 30 ± 3 nm for both hollow MSN and rattle-type MSN, and the average core diameter in rattle-type MSN is around 260 ± 10 nm. The large mesopore channels in rattle-type and hollow MSNs are clearly visualized *via* SEM images and N_2_ adsorption/desorption characterizations (Fig. S1), which means the air in the cavities of rattle-type and hollow MSNs can be emptied and replaced by degassed water. Additionally, comparing the measured and theoretical loading content of degassed water (Table S1) demonstrates that there is no gas in the cavities of rattle-type and hollow MSNs. Therefore, the potential influences of gas on the resonant frequency (ƒ*_R_*), nonlinear backscattering and impedance acoustic of either hollow or rattle-type MSNs, can be excluded without doubt. Noticeably, B fundamental imaging (BFI) mode, rather than other modes, was employed in all ultrasound imaging expriments, since BFI mode can truthfully reflect the imaging capability of UCAs, simultaneouly excluding the interferences from bubbles-induced harmonic and artifacts under contrast harmonic imaging (CHI) mode and color Doppler imaging (CDI) mode, respectively^11^,^14^,^22^,^23^,^24^,^25^.

Under BFI mode with a broadband excitation frequency centered at 10 MHz (Fig. 1c_0_–c_3_ and 1d), it is found that rattle-type MSN (126) as UCAs demonstrates much larger gray value than those of s-SiO_2_/h-SiO_2_ (79) and hollow MSN (86), and the percentages of the secondary interface's contributions for gray value are 32% = (126−79)/126*100% and 37% = (126−86)/126*100% relative to hollow MSN and solid structures, respectively. The average gray ratio of rattle-type MSN to s-SiO_2_/h-SiO_2_ (1.60) shows a similar value to that of rattle-type MSN to hollow MSN (1.47), thus accordingly, the sound intensity ratios of rattle-type MSN to s-SiO_2_/h-SiO_2_ and to hollow MSN are 2.56 and 2.16, respectively, suggesting the identical number of interface in both s-SiO_2_/h-SiO_2_ and hollow MSN is responsible for the similar imaging outcomes. Under BFI mode, both reflection and scattering signals corporately contribute to enhancing ultrasound imaging, and especially the latter contribution is usually dominant^18^. Therefore, both will be focused, respectively, when clarifying why rattle-type MSN shows more excellent capability of enhancing ultrasound imaging than either hollow MSN or s-SiO_2_/h-SiO_2_ in the following text.

### Proposal of double-scattering/reflection in a single nanoparticle

Before analysis, some probable interferences should be excluded so as to effectively and reliably find out the reasons. Firstly, the probable interferences from all the differences in particle size, particle concentration and composition can be excluded^4^, since the three nanostructures share the identical particle size, particle concentration of 2.12 × 10^8^ per ml and composition. As to the interparticle multiple-scattering, the much shorter average particle separation (17 μm) than the wavelength (150 μm) of ultrasound waves and the linear relation between average gray value and particle concentration (in other words, square relation between sound intensity and particle concentration) within 2.65 × 10^8^ per ml determines the presence of interparticle multiple scattering (Fig. S2A)^26^,^27^,^28^, smilar to hollow MSN and s-SiO_2_/h-SiO_2_ (Fig. S2B, C). However, since the three different silica nanostructures share the same particle number, the contributions from interparticle multiple scattering for the three different nanostructures are approximately identical, which means interparticle multiple scattering is not the cause of imaging capability difference for the three different silica nanostructures. Therefore, when investigating what induced the imaging capability difference of the three different silica nanostructures, the interparticle multiple scattering will not be taken into consideration. Moreover, in the following reflection and scattering acoustic measurements, the much less particle concentration than that in the ultrasound iamging evaluations can exclude the interparticle multiple-scattering, which will be detailedly explained in the following part. Additionally, standing waves between reflection waves and incident waves in a single rattle-type nanoparticle can also be neglected, since scattering is in all directions^29^. Taken all above together, it is found that such a superior capability of enhancing ultrasound imaging using rattle-type MSN against other two nanostructures can be exclusively attributed to its structural difference, namely, the number of interface.

**Figure 2:**
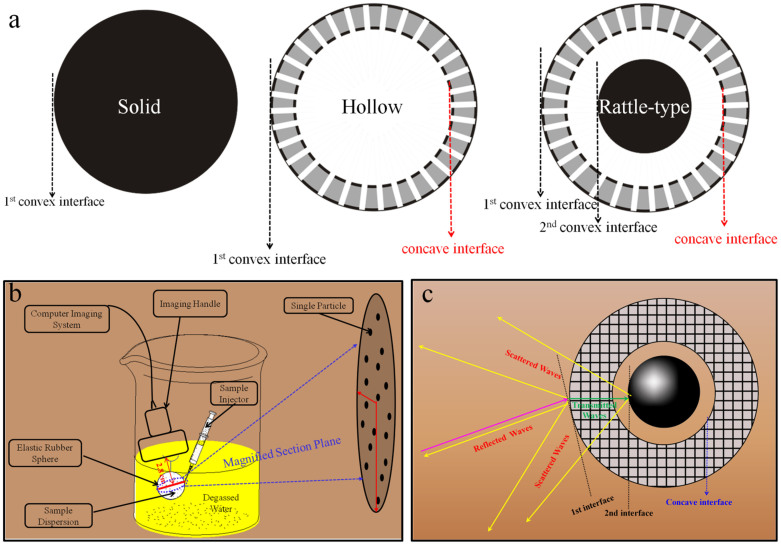
Sketch maps of three silica-based nanostructures and the mechanism of double-scattering in a single rattle-type MSN nanopsrticle. (a) The schematic illustration of possible interfaces in three typical types of nanostructures of solid, hollow and rattle-type. In detail, only one convex interface exists in solid nanostructure (left); one concave interface and one convex interface exist in hollow nanostructure (middle); and, two convex interfaces and one concave interface exist in rattle-type nanostructure (right). (b) Schematic illustration of the experimental apparatus of conducting ultrasound imaging; and (c) Schematic illustration of double-scattering/reflection in a single rattle-type nanoparticle. The contribution from concave interface in enhancing ultrasound imaging is trivial, hence can be neglected when investigating UCAs. Notes: The elastic rubber sphere was used as a container to load as-measured samples.

The mechanism of double-scattering/reflection in a rattle-type MSN nanoparticle can be seen in Fig. 2c. Since rattle-type MSN presents a specific structure of two contributing interfaces (Fig. 2a, c), after the incident ultrasound waves (denoted in purple) are scattered and reflected by the 1^st^ interface and the transmitted ultrasound waves (denoted in green) can be further scattered or reflected by the 2^nd^ interface, twice scattering/reflection in a single rattle-type nanoparticle in total will take place. For comparison, either hollow MSN or s-SiO_2_/h-SiO_2_ contains only one contributing interface (Fig. 2a), consequently generating only once scattering/reflection in a single nanoparticle, thus both of them produce the approximately similar contrast and gray value, but much lower than that of rattle-type MSN. To further demonstrate the importance of two scattering/reflection interfaces, a parallel experiment was conducted, wherein the particle concentration of rattle-type MSN is half of that of hollow MSN or s-SiO_2_/h-SiO_2_ (2.12 × 10^8^/ml) so as to guarantee the identical number of interfaces. In Fig. S3, the average gray value (66) of rattle-type MSN is slightly lower than that of hollow MSN (86) or s-SiO_2_/h-SiO_2_ (77) due to the smaller 2^nd^ interface than the 1^st^ one, nevertheless, it determines that the contributions of the second interface for gray value in rattle-type MSN are 35% = (66−86/2)/66*100% and 42% = (66−77/2)/66*100% for hollow MSN and solid s-SiO_2_/h-SiO_2_, respectively, which are in agreement with that obtained with the same particle concentration.

### Simulation calculations and acoustic measurements concerning the reflection contributions

Fig. 2b is the schematic illustration of the experimental system for ultrasound imaging. Generally, when referring to the reflection contribution, the population of nanoparticles in the cross plane (in the magnified area plane of Fig. 2b) of elastic rubber bag can be investigated as a whole, since reflection usually occurs at an interface of large area. Therefore, under the same particle concentration, the rattle-type MSN contains two layers of reflection interfaces in the cross plane, while either s-SiO_2_/h-SiO_2_ or hollow MSN contains only one layer of reflection interface in the cross plane. In the common reflection equation 3, the ratio of reflection intensity to incident intensity is defined as Intensity Reflection Coefficient (*IRC*) which can evaluate reflection contribution^30^. Moreover, it can be found that reflection is only dependent on material density and sound velocity (in other word, acoustic impedance), and independent of particle size. According to equation 3, for rattle-type MSN, twice reflection occur at both the 1^st^ interface and the 2^nd^ interface, so the *IRC* values for the 1^st^ and 2^nd^ interfaces are calculated to be 0.613 and (1 − 0.613) × 0.613, respectively, and the sum of reflection contributions, *IRC* (sum), is 0.613 + (1 − 0.613) × 0.613 = 0.850. While for hollow MSN and s-SiO_2_/h-SiO_2_, since only single-reflection occurs at the 1^st^ interface, *IRC* (the 1^st^ interface) values remain 0.613, and thus, the ratios of the reflection contributions of rattle-type MSN to s-SiO_2_/h-SiO_2_ and rattle-type MSN to hollow MSN are 1.39.

**Figure 3:**
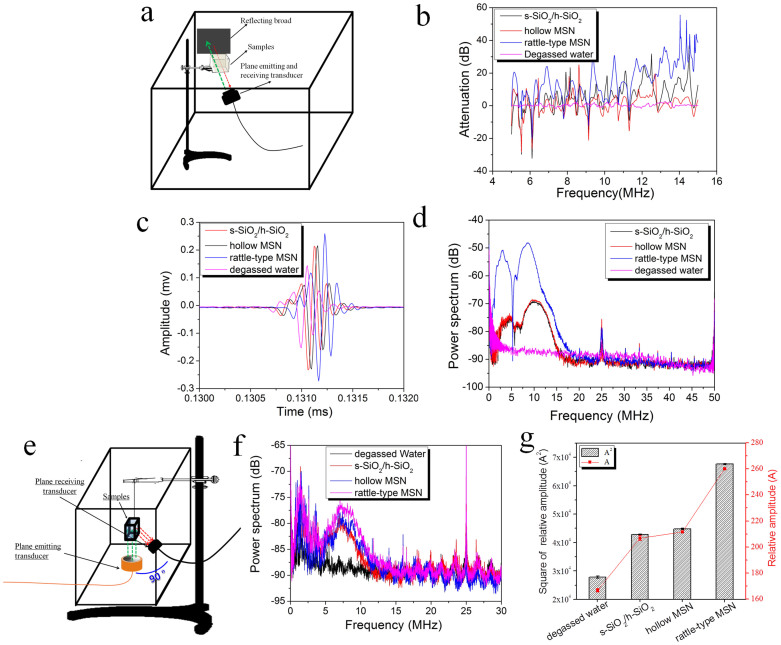
Acoustic measurements of reflection signals and scattering signals. (a) The device schematics of measuring reflection signals; and (b) Measurement results of reflection contributions: attenuation-frequency characteristic of sound in suspensions containing three different nanostructures of the same particle concentration of 2.65 × 10^7^/ml, respectively, which were obtained from the FFT transformation of c; (c) Time-amplitude trace and (d) Actual reflection spectra of the three different nanostructures. Notes:The same plane transducer (transducer centered at 10 MHz) with a 3-dB bandwidth was employed to emit and receive signals. (e) The device schematics of measuring scattering signals. (f) Actual scattering signal spectra of different samples of the same particle concentration of 2.65 × 10^7^/ml and (g) Measurement results of their scattering contributions: relative amplitude (A) and its square (A^2^) of scattered signals in PBS solution and dispersion containing three different nanostructures. Notes: A plane transducer (10 MHz) with a 3-dB bandwidth was employed to emit incident ultrasound waves, and another identical plane transducer was employed to receive the scattered signals.

Additionally, another evidence of much enhanced ultrasound imaging of rattle-type MSN relative to either s-SiO_2_/h-SiO_2_ or hollow MSN was obtained by measuring acoustic reflection signals. Fig. 3a schematically shows the measurement device for detecting reflection signals. The attenuation traces (Fig. 3b) and actual frequency spectra (Fig. 3d) of the three UCAs convincingly indicate that rattle-type MSN has led to the strongest reflection spectra and consequently the largest attenuation value in almost the whole of frequency range among the three nanostructures. The oscillations in the traces of Fig. 3b may be attributed to the particle migration under acoustic irradiation force. Furthermore, according to the amplitude-time trace (Fig. 3c), the amplitudes of the reflection signals received from s-SiO_2_/h-SiO_2_, hollow MSN and rattle-type MSN are 0.222 mV, 0.223 mV, 0.268 mV, respectively. It is well known that the square of the amplitude is proportional to gray value, so the ratios of sound intensity of rattle-type MSN to s-SiO_2_/h-SiO_2_ and to hollow MSN can thus be calculated to be 1.47 and 1.44, respectively. The two values are approximately equal to the vaule (1.39) obtained *via* above simulation calculations but lower than that (2.56 and 2.16) deriving from the calculations for their corresponding gray values in Fig. 1d. This result can be attributed to the employed much lower particle concentration (2.65 × 10^7^/ml) during reflection acoustic measurement than that (2.65 × 10^8^/ml) employed in ultrasound imaging evaluations exclude the interferences from interparticle multiple scattering. However, the larger IRC of rattle-type MSN than either s-SiO_2_/h-SiO_2_ or hollow MSN *via* both therotical calculation and reflection acoustic measurement still demonstrate the occurrence of double backscattering in a single rattle-type MSN particle. The peak at ~25 MHz suggests a systematic error, as a similar peak does in Fig. 3f.

### Simulation calculations and acoustic measurements concerning the scattering contributions

Since the interparticle multiple-scattering under the particle concentration of 2.65 × 10^7^/ml can be neglected, equations 4 and 5 as modified rayleigh scattering equations that can perfectly fit fundamental scattering in long wavelength scattering theory^4^,^27^, are believed reliable to investigate linear scattering contributions^29^,^31^. According to equation 6, the values of the resonant frequency, ƒ*_R_* of solid, hollow and rattle-type structures are +∞, 16.4 GHz and 24.2 GHz, respectively, far higher than that of the frequency, ƒ (10 MHz) applied in ultrasound imaging experiments and acoustic measurements, which theoretically determines no nonlinear harmonics. Therefore, the above-mentioned scattering should belong to the linear fundamental scattering, and the influence of nonlinear harmonic signals that will occur only when gas bubbles exists in the cavities of hollow or rattle-type MSN, or when ƒ*_R_* of the three structures is close to ƒ to induce resonance, can be neglected beyond doubt. Simultaneously, it also suggests the reason why s-SiO_2_/h-SiO_2_ and hollow MSN share the approximately identical ultrasound imaging outcomes despite the presence of structure differences for s-SiO_2_/h-SiO_2_ and hollow MSN.

According to equation 6, it is found that ƒ*_R_* is closely associated with shell thickness and elasticity. Generally speaking, calcination treatment can increase the compactness of Si-O-Si network and reduce the structural elasticity. Therefore, it is not difficult to understand that both uncalcined hollow MSN and rattle-type MSN *via* our one-pot synthesis strategy perform much better in enhancing US imaging contrast than calcined ones under BFI mode (Fig. S4)^21^.

Since the ƒ*_R_* values of the three silica-based nanostructures are far larger than the applid ƒ(10 MHz)^17^,^27^,^32^, slight increment or reduction of ƒ*_R_* will not obviously change the influence of ƒ*_R_* on the intensity scattering coefficient (ISC), according to equations 4 and 5, and the influence of r as the sole independent variable on scattering, is dominant. *ISC* is proportional to the scattering cross-section (σ_s_), and further proportional to the six power of the radius of scattering particles. Fig. 3e shows the device schematic of detecting scattering signals. From the related acoustic measurements of scattering signals (Fig. 3f), it is found that rattle-type MSN indeed generates the highest intensity of scattering signals among the three UCAs, and the square of the amplitude of relative scattering (A^2^) using s-SiO_2_/h-SiO_2,_ hollow MSN and rattle-type MSN (Fig. 3g) as UCAs are 42927, 44785 and 67745, respectively. Hence, the scattering contribution ratios of rattle-type MSN to s-SiO_2_/h-SiO_2_ and rattle-type MSN to hollow MSN are 1.58 and 1.51, respectively, which convicingly demonstrate the occurrence of double backscattering in a single rattle-type MSN particle. As stated in reflection acoutic measurement, the lower values (1.58 and 1.51) obtained *via* scattering acoustic measurement than that (2.56 and 2.16) deriving from the calculations for their corresponding gray values in Fig. 1d can be attributed to the presence of interparticle multiple scattering in ultrasound imaging evaluations but the absence of interparticle multiple scattering in scattering acoustic measurement is due to the difference of particle concentration. Noticeably, the reason of the peaks occurring at approximately 7.5 MHz rather than the sonication frequency of 10 MHz may be the fact that the actual center frequency of the transmitting transducer is lower than standard value (10 MHz) given by the manufacturer of transducer.

**Figure 4:**
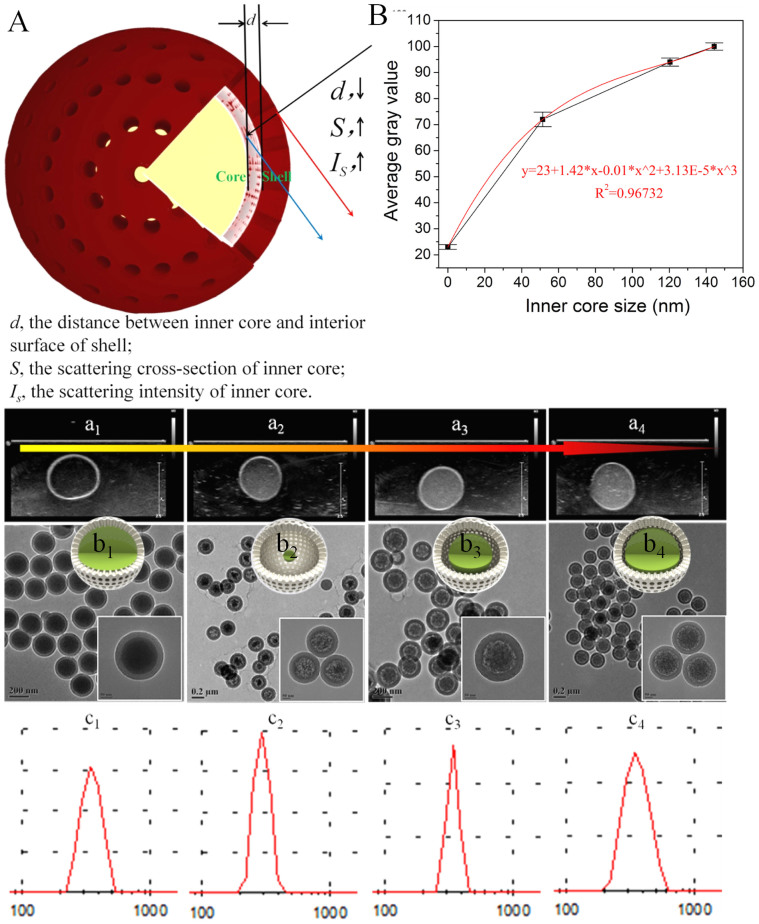
The influence of inner core size in rattle-type MSN on ultrasound imaging. (A) Schematic illustration of the relationship between *I_s_* and *d*. (B) Measured average gray values. (a_1_–a_4_) Ultrasound images under B fundamental imaging mode at the emission center frequency of 10 MHz. TEM images (b_1_–b_4_) and DLS data (c_1_–c_4_) of s-SiO_2_/h-SiO_2_, rattle-type MSN-1, rattle-type MSN-2 and rattle-type MSN-3, respectively, and the insets in (b_1_–b_4_) are their respective **3-D** model.

As indicated above, the most important feature of rattle-type MSN against other two nanostructures in enhancing ultrasound imaging lies in the presence of the 2^nd^ spherical interface. Therefore, the influence of the 2^nd^ spherical interface area on enhancing ultrasound imaging needs to be further investigated. Herein, a batch of rattle-type mesoporous silica nanostructures with tunable inner core sizes have been designed and synthesized. The typical TEM images (Fig. 4b_1_–b_4_) show the diameter of inner core in rattle-type MSN (230 nm) can be varied from 0, to 50 nm, to 120 nm and till to 145 nm, correspondingly named as s-SiO_2_/h-SiO_2_, rattle-type MSN-1, rattle-type MSN-2, and rattle-type MSN-3. The high monodispersity and narrow size distribution can be further demonstrated by DLS (Fig. 4c_1_–c_4_). Similarly, other potential influence factors, such as interparticle multi-scattering, particle concentration, size, composition, *et al*., can also be excluded.

In *in vitro* ultrasound imaging results under BFI mode (Fig. 4a_1_–a_4_), as can be found, with the increase of inner core size, the corresponding average gray value increases accordingly from 23, to 73, 95 and 100 (Fig. 4B). Therefore, it is obtained that with the decrease of gap (d) between inner core and outer shell, *i.e.*, the radius (r) of inner core increase, thus the σ_s_ of the 2^nd^ scattering interface accordingly increase, consequently elevating the backscattering intensity of the 2^nd^ interface^17^,^27^. Clearly, rattle-type MSN-3 achieves the strongest attenuation signals (Fig. S5) at around 10 MHz since it has the largest σ_s_ of the 2^nd^ interface. Additionally, a cubic fitting formula depicting the relationship between average gray value and the radius of inner core (Fig. 4B) can be obtained. In this plot, since the real intensity (I, unit: W/m^2^) is positively proportional to the square of gray value, the power exponent of radius in correlation to average gray value conforms to equation 4, and the other variants (r, r^2^) is probably attributed to absorption attenuation, thermal transport^16^,^17^,^33^, and unexpected high & low order harmonics^27^.

### The universality demonstration of this structure design concept

In order to confirm the feasibility and universality of the proposed structure-based design concept in guiding new generation UCAs' design, firstly, a double-shelled hollow mesoporous silica nanostructure (DHMSN) has been fabricated. Although the inner core structures of rattle-type MSN-3 and DHMSN are different, (solid silica sphere in rattle-type MSN-3, while hollow sphere in DHMSN), the two nanostructures share the identical particle size, number of interfaces, thickness of outer shell, and size of inner core. Interestingly, rattle-type MSN-3 and DHMSN have achieved approximately equal gray value and attenuation value at 10 MHz, refelecting the presence of double-scattering/reflections in the two nanostructures (Fig. S6).

Moreover, a heterogeneous rattle-type nanostructure (rattle-type Au-MSN) with Au nanoparticles as inner core was fabricated^21^. Such rattle-type Au-MSN can also provide two convex interfaces to generate double-reflecting/scattering in a single nanoparticle. As expected, rattle-type Au-MSN indeed performs much better in intensifying ultrasound imaging than hollow MSN (Fig. S7). Similarly, according to this structure design strategy, a rattle-type Fe_2_O_3_@HMSN (US/MR dual-mode probes) has been designed and synthesized, wherein Fe_2_O_3_ not only serves as MR contrast agent to enahnce MRI contrast (Fig. S8e–f), but also as a scattering/reflection interface to improve ultrasound imaging contrast (Fig. S8a–d). Additionally, The ultrasonic detection limits of the three different nanostructures were also evaluated, since high detection sensitivity is necessary, especially at diagnostic frequency^34^. In Fig. S9, the ultrasonic detection limit of rattle-type MSN is between 0.015 and 0.0075 mg·ml^−1^ in mass or between 0.25 mM and 0.13 mM in molar, which is much lower than that of hollow MSN and s-SiO_2_/h-SiO_2_, implying that rattle-type MSN has higher ultrasound sensitivity than solid or hollow MSN owing to its double-scattering/reflection interfaces.

### Intracellular and *in vivo* ultrasound imaging using rattle-type MSN

Biocompatibility is a crucial factor for UCAs in the practical biomedical application, and it has been well demonstrated that mesoporous silica nanoparticles have a high biocompatibility^35^,^36^,^37^,^38^. Herein, both *in vitro* cytotoxicity and *in vivo* blood and tissue toxicities of rattle-type MSN were evaluated, and results show no evident toxicities, confirming good biocompatibility of rattle-type MSN (Figs. S10–S12).

**Figure 5:**
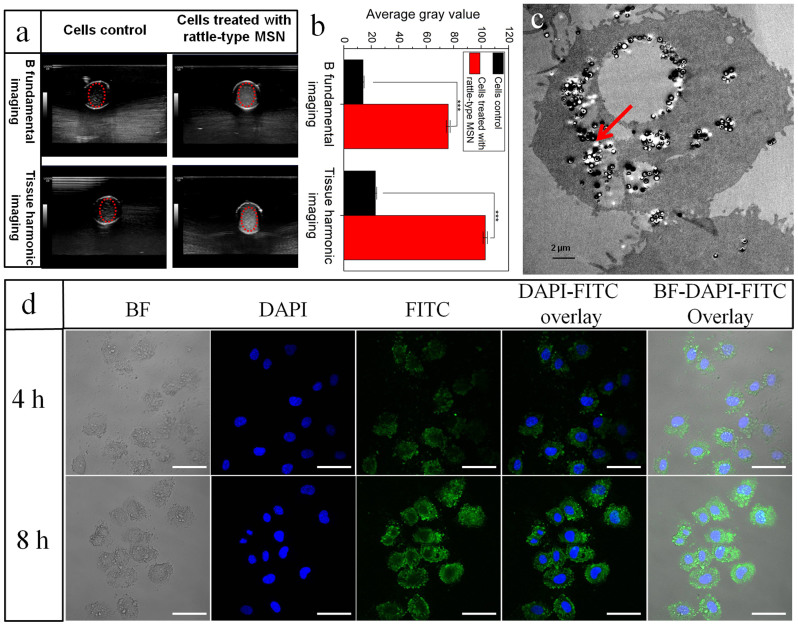
*Intracellular* ultrasound imaging of rattle-type MSN nanoparticles. (a) Cellular ultrasonic images of L929 intact cells with and without uptaking a large amount of rattle-type MSN particles under B fundamental imaging (BFI) and tissue harmonic imaging (THI) modes; (b) The corresponding average gray values; (c) Bio-TEM images of L929 cells after uptaking rattle-type MSN particles; (d) Internalization test of rattle-type MSN nanoparticles with a concentration of 200 ug·ml^−1^ by L929 cells in different time intervals (4 h and 8 h). Notes: *** represents significant differences in average gray value by comparing wells alone with cells treated with rattle-type MSN at P ≤ 0.001. BF represents bright field, FITC represents FITC-labeled rattle-type MSN, and the scale bar is 50 μm.

Afterwards, the ultrasound imaging capability of rattle-type MSN nanoparticles for intact cancer cells is evaluated. It is clearly observed that a large number of rattle-type MSN nanoparticles are endocytosed by cancer cells from confocal images (Fig. 5d) and bio-TEM image (Fig. 5c), in detail, quantitatively up to 2.64 mg can be uptaken by 6.0 × 10^8^ L929 cells after incubation for 24 h (113 rattle-type MSN nanoparticles per cell), which provides the great opportunity of intracellular ultrasound imaging. As expected, for rattle-type MSN, much larger contrast and higher average gray values of cells than control group are obtained (Fig. 5a, b), confirming the excellent capability of rattle-type MSN nanoparticles in intensifying cellular-level ultrasound imaging.

**Figure 6:**
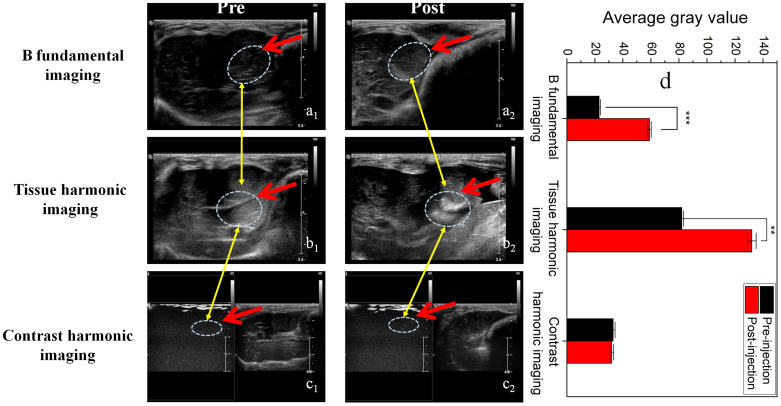
*In vivo* ultrasound imaging of rattle-type MSN nanoparticles. (a_1_–a_2_) *In vivo* ultrasound images of VX2 liver tumor in the rabbit model under B fundamental imaging (BFI) mode before (a_1_) and after (a_2_) injecting rattle-type MSN particles of the particle concentration of 10^8^ orders; (b_1_–b_2_) *In vivo* ultrasound images of VX2 liver tumor in rabbit model under tissue harmonic imaging (THI) mode before (b_1_) and after (b_2_) injecting rattle-type MSN particles at the particle concentration of 10^8^ orders; (c_1_–c_2_) *In vivo* ultrasound images of VX2 liver tumor in the rabbit model under contrast harmonic imaging (CHI) mode before (c_1_) and after (c_2_) injecting rattle-type MSN particles of the particle concentration of 10^8^ orders; (d) Enhanced average gray values of before and after injecting rattle-type MSN particles under BFI, THI and CHI modes in the zones of interest (circled by dotted ellipse and indicated by red arrows). Notes: ** and *** represent significant differences in average gray value by comparing before injecting rattle-type MSN and after injecting rattle-type MSN at P ≤ 0.01 and P ≤ 0.001, respectively.

Fig. 6 shows *in vivo* ultrasound imaging results on the grafted VX2 solid liver tumor of rabbits under different imaging modes. Under BFI mode, a distinctive increment in the contrast of *in vivo* ultrasound images after administrating rattle-type MSN nanoparticles *via* subcutaneous injection puncture (Fig. 6a_1_, a_2_) is observed. Quantitatively, the average gray value (calculating the circled zones within dotted line, Fig. S14) also accordingly increases from 24 to 64, and the increment is much larger than that using s-SiO_2_/h-SiO_2_ and hollow MSN, which directly confirms the excellent *in vivo* imaging performance of rattle-type MSN. In addition, under tissue harmonic imaging (THI) mode^39^,^40^, great increases in contrast and average gray value (Figs. 6b_1_, b_2_ and S15) both *in vitro* and *in vivo* can be attributed to the migration of rattle-type MSN nanoparticles driven under sound pressure and the variation of sound velocity^41^,^42^.

As discussed above, owing to the absence of gas in the cavity of rattle-type MSN and the considerable difference between ƒ*_R_* (16.4 GHz) of rattle-type MSN and applied ƒ (10 MHz), it is assured of no nonlinear backscattering. Therefore, under CDI mode under which the harmonic component from the tissues can be subtracted out, and the received signals mainly come from the second harmonic of UCAs, no nonlinear harmonic signals (Fig. 6c_1_, c_2_) emerge.

## Discussion

To improve ultrasound imaging quality, especially for inorganic-based UCAs, increasing the number of scattering/reflection interfaces to improve the utilization of ultrasound waves in ultrasound imaging will be a feasible and important solution. Based on this, a novel structure-based design concept of UCAs (double-scattering/reflection in a single nanoparticle) has been proposed for the first time. Depending on the two contributing interfaces, double-scattering/reflection occurs in a single rattle-type MSN nanoparticle. Therefore, from the perspectives of ultrasound imaging evaluations, together with simulation calculation and acoustic measurements, rattle-type MSN performs much better in enhancing ultrasound imaging than solid and hollow nanostructures with only one interface. The σ_s_ (attenuation and cross-section area) is the determinant of influencing ultrasound imaging, and is proportional to either scattering intensity and or reflection intensity. Therefore, with the increase of inner core size in rattle-type MSN, the σ_s_ of the 2^nd^ interface increase, and accordingly, the imaging outcome increase.

As a universal strategy, this structure-based design strategy can be extended to guide the design and fabrication of other UCAs with different compositions but similar structures, such as DHMSN, rattle-type Au-MSN and Fe_2_O_3_@HMSN *et al*. For example, it is expected that double-layer microbubbles will more evidently enhance ultrasound imaging. Besides *in vitro* evaluations, the *cellular-level* and *in vivo* ultrasound imaging also demonstrate the excellence of double-scattering/reflection in a single rattle-type MSN.

In summary, the structure-based design concept of UCAs has been proposed to overcome the bottleneck of low utilization of ultrasound waves that usually occur in the conventional composition design strategy. Three different silica-based nanostructures have been prepared and used as ideal models to theoretically and experimentally exemplify this novel design concept by large number of experimental researches, detailed theoretical calculations as well as acoustic measurements. Furthermore, the direct influence of inner core size of rattle-type MSN on ultrasound imaging performances also demonstrates the occurrence of double-scattering/reflection in a single rattle-type MSN nanoparticle. More importantly, this structure-design concept can extend to guide the design of many other UCAs, and it can also find many interesting and useful application fields, such as intact cells' imaging and *in vivo* tumor imaging. It is assured that this novel design strategy of double-scattering/reflection in a single particle should be a valuable method in guiding the design of new generation UCAs.

## Methods

### Materials

Tetraethoxysilane (TEOS,A.R), ammonia solution(NH_3_·H_2_O) (25~28%, A.R), and sodium carbonate anhydrous(Na_2_CO_3_,A.R) were obtained from Shanghai Lingfeng Chemical Reagent Co.LTD. Ethyl alcohol absolute (EtOH) was obtained from Shanghai Zhenxing No.1 Chemical Plant. 3-Aminopropyltriethoxysilane (98%, APTES) was purchased from J&K Scientific LTD. Deionized water was used in all experiments. Cetyltrimethyl Ammonium Bromide (CTAB) was purchased from Sigma-Aldrich. Fluorescein isothiocyanate isomeric (FITC, 90%) was purchased from ACROS ORGANICS.

### Characterization

FETEM (field emission transmission electron microscopy) analysis of all samples were conducted with a JEM 2100 F electron microscope operated at 200 kV. Nitrogen adsorption–desorption isotherms of the three samples (s-SiO_2_/h-SiO_2_, hollow MSN and rattle-type MSN)were measured at 77 K on a Micromeritics Tristar 3000 analyzer. The pore-size distributions of all samples were calculated using adsorption isotherm branches by the BJH method, and their pore volumes and specific surface areas were calculated by using BJH and BET methods, respectively. The ultrasound images of all samples were obtained from Philips IU22, and their average gray values was obtained *via* image processing software, SONOMATH—DICOM. Particle size distributions of all samples with diluted concentrations were measured by dynamic light scattering (DLS) on Malvern Nano-ZS90. Live cellular internalization studies of rattle-type MSN were performed with Olympus confocal microscopy. Cell viability of rattle-type MSN using flow cytometry method was conducted on BD FACSCalibur. The *in vitro* T1-weighted MRI measurements was performed on a 3.0 T MRI instrument (GE Signa 3.0 T), and a T1-weighted FSE-XL/90 sequence (Parameters: TR/TE = 1000, 2000, 3000, 4000/7.9 ms; slice thickness = 2 mm; field of view [FOV] = 18 cm^2^; FOV = 18 cm; NEX = 2; space = 0.5 mm; matrix = 128 × 128; coil = QUADKNEE) was employed as the pulse scanning sequence.

### Preparation of s-SiO_2_/h-SiO_2_, rattle-type MSNs with s-SiO_2_ as inner core and hollow MSNs

In a typical process, 71.4 ml ethyl alcohol (EtOH) and 10 ml deionized water and 3.14 ml aqueous amnonia were mixed with each other at 30°C for 30 min. Then 6 ml tetraethyl orthosilicate (TEOS) was added quickly into the mixture above and reacted for 40 min at 30°C, after that, another mixture consisting of 5 ml TEOS and 2 ml (3-Aminopropyl)triethoxysilane (APTES) was added quickly and continued reacting for another 75 min, forming s-SiO_2_/h-SiO_2_ core/shell structure. The suspension was centrifuged and re-dispersed into 50 ml Na_2_CO_3_ (0.6 M) solution, followed by a hydrothermal treatment at 80°C for 15 min, forming rattle-type MSN. If the etching time was extended to 40 min, hollow MSN nanoparticles were produced. Finally, the suspension was centrifuged and washed with water for three times and dried under vacuum.

### Preparation of APTES-FITC

Fluorescein isothiocyanate (FITC) (5.5 mg) was dissolved in a solution containing ethanol (3 ml) and APTES (12 μl) and then stirred for 4 h, followed by preserving under air seasoning.

### Preparation of s-SiO_2_/h-SiO_2_, rattle-type MSN-1, rattle-type MSN-2, rattle-type MSN-3 and DHMSN

Firstly, SiO_2_ spheres were prepared. In a typical synthesis, 3 ml of TEOS were rapidly added into a mixture of ethanol (35.7 ml), deionized water (5 ml), and ammonium aqueous solution (25–28%, 1.57 ml). The mixture was then stirred at room temperature for 1 hr, resulting in the formation of a white silica colloidal suspension, and then the particles were centrifugally separated from the suspension and washed with deionized water and ethanol. The silica particles were re-dispersed in denoized water (14 ml) for use.

Secondly, above-prepared silica suspension (2 ml) was diluted and dispersed with 8 ml deionized water under ultrasonication, forming SiO_2_, and then added into a mixed solution consisting of hexadecyltrimethylammonium bromide (CTAB) (75 mg), deionized water (15 ml), ethanol (15 ml) and aqueous ammonia (0.275 ml) and stirred for 30 min. Another mixture containing TEOS (0.125 ml) and FITC-APTES (0.15 ml) was added into the suspension above, and stirred for 2 h at 30, forming core/shell structure s-SiO_2_/h-SiO_2_. After that, the s-SiO_2_/h-SiO_2_ particles were dispersed into 10 ml Na_2_CO_3_ (212 mg) aqueous solution and then etched at 50°C for 10 h, at 80°C for 15 min, or at 70°C for 3 h, obtaining rattle-type mesoporous silica nanostructures (MSN) with different sizes of inner cores, corresponding to rattle-type MSN-1, rattle-type MSN-2 and rattle-type MSN-3, respectively. If the etching conditions were fixed at 80°C for 30 min, double-shell hollow mesoporous nanostructure (DHMSN) would be formed. Finally, s-SiO_2_/h-SiO_2_, rattle-type MSN with different sizes of inner cores and DHMSN were collected by centrifugation and washed with water for three times, and then dried under vacuum.

### Calculation of particle volume fractions (β) in total dispersion

The volume of a single rattle-type MSN nanoparticle can be calculated:
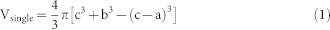
a: the shell thickness of rattle-type MSN;

c: the integral particle size of rattle-type MSN;

b: the solid silica inner core size in rattle-type MSN;

V_single_: the single particle volume of each rattle-type MSN nanoparticle;

Then, the β value was obtained according to the following formula.
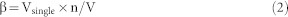
n: the total particle number of rattle-type MSN nanoparticles;

V: the given dispersion volume.

### *In in vitro* ultrasound imaging

The as-synthesized rattle-type MSN of a certain concentration was placed and sealed in an elastic rubber bag, and then the bag was immersed into a cistern full of PBS solution. The detector of ultrasound imaging instrument was fixed on the position with spacing of 2.8 cm from the elastic rubber bag filled with undetermined sample solution, especially 2.8 cm spacing is the most optimal value^17^. The received signals were converted into images exhibited on the screen. The average gray values can be obtained from an image processing software, SONOMATH—DICOM. In all *in vitro* experiments, the broadband excitation frequency centering at 10 MHz with a bandwidth of 8–12 MHz was employed, The parameters of Philips IU 22 are as follows: the transducer is L12-5, the mechanical index (MI) is 0.6, and other setting parameters are that software: QLAB, mode: SmPrt Sup, frame frequency (FR): 32 Hz.

For the critical limit values of rattle-type MSN in mass concentration and molar concentration, the operation details are identical with above measurements, and the employed strategy is gradually dilution to detect the C_1_ and C_0_, between which C_limit_ will be placed.

### *In vitro* MR imaging assay

Aqueous dilutions of rattle-type Fe_2_O_3_@HMSN nanocomposite with the different Gd concentrations were placed in a series of 1.0 ml tubes for T2-weighted MR imaging. The Fe atom content of rattle-type Fe_2_O_3_@HMSN was determined by inductively coupled plasma atomic emission spectrometry (ICP-AES). The T2 values were recorded at different concentrations and plotted as 1/T1 *vs.* molar concentration of Fe atoms. And then the slope of this line provides the molar relaxivity r2.

### Reflection and scattering measurement

In reflection measurement experiment, the transducer centering at 10 MHz with a 3-dB bandwidth was employed to simultaneously emit and receive ultrasound, and it was fixed on wall of the large container full of degassed water (60 cm × 60 cm × 60 cm) perpendicular to one surface of the sample pool. Another end of the transducer was linked with oscilloscope that communicates with the computer to record, analyze and address the reflected signals *via* certain softwares. A total reflection panel was placed behind the sample pool to reflect the transmitted ultrasound waves from sample pool, and the sample pool was a sealed cubic holder (4 cm × 4 cm × 4 cm) with a tiny pore linked to injector tube and with a 2 cm × 2 cm square pore in all six planes of the sample pool covered with membrane that not only allows all incident waves in. The cubic sample holder was placed at between the transducer and the total reflection panel, and the 2 cm × 2 cm square pore was in line with the transducer to guarantee the successful measurement. The schematic image of the reflection measurement device can be seen in fig. 3a. Parameters of pulse emission receiver are that mode: P/E, high pass (HP): 1 KHz, low pass (LP): 35 MHz, attenuation: 10 dB, gain: 20 dB, pulse repetition frequency (PRF): 100 Hz, energy: 25 J. The parameters of high-speed data acquisition card are that sampling rate: 100 MS/s, sampling number: 50, sampling time: 2.0 s. The directly obtained data is time-amplitude trace, and the frequency spectrum and attenuation plot can be obtained *via* FFT transformation or other programmed transformation for time-amplitude trace *via* matlab programming.

As for the scattering measurement, except the number of transducers and their intersection angle, all experimental apparatus and test procedures were almost the same with those employed in the measurements of reflection signals. Herein, two identical transducers centering at 10 MHz with a 3-dB bandwidth were employed and perpendicularly fixed on two adjacent walls of the large container full of degassed water (60 cm × 60 cm × 60 cm), wherein one was employed as emitting transducer and another was employed as receiving transducer, and thus their intersection angle between incident and reflected waves is 90°. Herein, the total reflection panel was removed, and the cubic sample holder (4 cm × 4 cm × 4 cm) with a tiny pore linked to injector tube was placed at the focus of the two transducers so that the incident waves from one transducer were scattered by samples, and afterwards the scattered waves can be received by the another transducer. Noticeably, when replacing sample-for-test, the injector firstly pump out the last sample dispersion, and then injected the next sample dispersion, making sure the whole measurement apparatus would no longer move once they were set up. The schematic image of scattering measurement device can be seen in fig. 3e. Parameters of pulse emission receiver are as follows: P/E, high pass (HP): 1 KHz, low pass (LP): 35 MHz, attenuation: 10 dB, gain: 40 dB, pulse repetition frequency (PRF): 100 Hz, energy: 100 J. The parameters of high-speed data acquisition card are that sampling rate: 100 MS/s, sampling number: 50, sampling time: 2.0 s. During measurement, the amplitude of scattering signals can be directly obtained in the oscilloscope, and the frequency spectrum can be also obtained *via* programmed calculation for time-domain signal using matlab programming.

Before all measurements, the transducer was calibrated *via* measuring the distribution of sound pressure, and the obtained scattering or reflection signals can be transformed between each other *via* matlab software.

### Reflection equation

Theoretically, the *IRC* in whole incident sound intensity can be calculated using equation 3^30^, where *I_r_* and *I_i_* represent the reflection intensity and incident intensity, respectively. *Z_s_* = *ρ_s_*_·_*υ_s_* represents the acoustic impedance of special material (*ρ_s_* and *υ_s_* are the density of the reflector and sound velocity in the reflector, respectively); *Z* = *ρ*_·_*υ* is the acoustic impedance of medium (*ρ* and *υ* are density and sound velocity of the embedding medium, respectively). 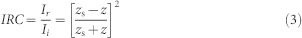


In degassed water medium, *ρ* and *υ* are common parameters, 1 g/cm^3^ and 1473 m/s, respectively, so Z = *ρ*·*υ* is 1.47 MRayls, In contrast, *ρ_s_* and *υ_s_* of silica are measured to be 2.2 g/cm^3^ *via* mercury intrusion method and 5500 m/s *via* measuring silica-assembed blocks, therefore, Z *s* = *ρ_s_*·*υ_s_* is 12.1 MRayls.

### Scattering equations

The *ISC*^4^,^27^ can be depicted with a modified equations 4 and 5, wherein, *I*_s_ represents the scattering intensity, and *I*_i_ represents the incident intensity; r is the radius of spherical interface; *σ*_s_ is the scattering cross-section of single scatterer (equation 5); L (≫r) is the fixed distance of detector from the scatterer in all experiments, generally is a constant; k = 2π/λ = wave number, where λ is the wavelength; *κ*_s_ is the adiabatic compressibility of the scatterer; κ is the adiabatic compressibility of the embedding medium; *ρ*_s_ is the density of the scatterer and *ρ* is the density of the embedding medium. Thus, *ISC* is closely associated with r, *κ*_s_ and *ρ*_s_, and for different silica-based nanostructures, *e.g.*, rattle-type MSN, hollow MSN and s-SiO_2_/h-SiO_2_, their *κ*_s_ and *ρ*_s_ are different.
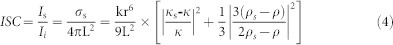

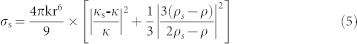
For inorganic nanoparticles without encapsulating free gas, their resonance frequency (ƒ*_R_*) can be obtained as follows:^17^,^31^
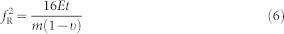
wherein, ƒ*_R_* denotes resonance frequency of shell; *f* is the frequency of the applied sound field; *m* is effective particle mass of single UCA nanoparticle; *t* is the wall thickness; *E* and *υ* are the Young's modulus and Poisson ratio of the scatterer, respectively^17^,^31^. For hollow silica nanoparticles *via* the hard template method of sol-gel chemistry, the *E* and *υ* are 18 GPa and 0.17, respectively^43^,^44^.

In this report, for s-SiO_2_/h-SiO_2_ (solid), its *t* is equal to the radius of the particle, 210 nm, and m is 4.81 × 10^−17^ kg per solid nanoparticle,and thus its *ƒ_R_* is 44.55 GHz. For hollow MSN particle, *t* is 30 nm, and m is 1.78 × 10^−17^ kg per hollow nanoparticle, and thus its *ƒ_R_* is 24.2 GHz, while for rattle-type MSN, *t* is 30 nm, but m is 3.89 × 10^−17^ kg per rattle-type nanoparticle, its ƒ*_R_* is 16.4 GHz.

### Cell Culture

Mouse fibroblast cells L929, brain capillaryendothelial cells (BCECs) and Hela cell lines were cultured and maintained in high glucose Dulbecco's modified Eagle's medium (DMEM) supplemented with 10% fetal bovine serum at 37°C in a fully humidified atmosphere of 5% CO_2_.

### MTT Assay

L929, BCECs and Hela cell lines were seeded in a 96-well plate at a density of 10^4^ cells per well in a 100 μL volume. Cells were further maintained at 37°C for 24 h and 48 h after treatment with rattle-type MSN. Cell viability was then determined using an MTT (3-(4,5-dimethylthiazol-2-yl)-2,5- diphenyltetrazolium bromide) assay (MTT cell growth assay kit, Chemicon, USA). The rattle-type MSN-treated cells were incubated with the MTT reagent for 4 h (viable cells are capable of metabolizing the MTT reagent, while dead cells are not), and 100 μl of DMSO was added to each well and incubated for 30 min, and the absorbance at 570 nm was read. Each concentration was repeated in triplicate, and the results are expressed as percentages.

### Flow cytometry

L929 cell lines were seeded in 6-well plates, with a cell number of 10^7^ orders. After the occupation percentage of cells was up to 80%, different mass concentrations of rattle-type MSN nanoparticles were added into the corresponding wells, and after incubation 24 h, the upper dead cells was collected, and simultaneously the bottom cells was harvested *via* 1 ml pancreatin (5%). Ultimately, all cells were stained by PI and annexin-V-FITC dyes, and instantly were detected *via* flow cytometry with two channels: red light channel, PI and green light channel, FITC.

### Confocal Luminescence Imaging

Here, the nanoparticles were incubated in DMEM with L929 cells for a given time, then the unbound nanoparticles were washed away, and the live cells were imaged using a confocal microscope. After incubation with 200 ug·ml^−1^ or 100 ug·ml^−1^ rattle-type MSN particles for 4 h and 8 h at 37°C, strong green fluorescence representing rattle-type MSN nanoparticles and blue fluorescence representing stained cell nuclei by DAPI with high signal-to-background ratio was observed in the cells, and the green fluorescence intensity after incubation with nanoparticles for 8 h was stronger than that after incubation with nanoparticles for 4 h under similar imaging conditions. Meanwhile, the fluorescence confocal images showed the presence of healthy and round nuclei, which could be excited at 405 nm when stained blue by using 4,6-diamino-2-phenylindole (DAPI). DAPI is a blue fluorescent dye known to complex with double stranded DNA in nuclei. Green fluorescence from rattle-type MSN nanoparticles was well detected in the cell bodies of the L929 cells. The DAPI and green-fluorescent-dye-excited images were recorded at the same focal depth. Confocal luminescence imaging was performed with an Olympus FV1000 laser scanning confocal microscope and a 60 × oil-immersion objective lens. The L929 cells, seeded in a 35-mm glass-bottomed culture dish, were washed with PBS buffer and then incubated with 200 μg·ml^−1^ of rattle-type MSN for 4 h and 8 h at 37°C. After being washed three times with PBS, the cells were then stained with DAPI solution for 15 min, another three times wash was conducted to remove the reluctant DAPI solution, and after that 500 μL buffer A solution was added to conduct confocal luminescence imaging.

### Hemolysis assay

Fresh human blood anticoagulated with EDTA anticoagulant was obtained from Shanghai (Red Cross) Blood Center, and the hemolytic and coagulation assays were approved by the ethics committees of Shanghai (Red Cross) Blood Center. Fresh human blood was centrifuged for 10 min to remove plasma at 3000 rpm. After sterilization, human red blood cells were collected *via* isotonic washing with PBS for 5 times, and then were diluted into 10 fold with PBS for use. 300 ul red blood cells dispersion was added into the following groups: (1) 1.2 ml ultrapure water (negative control), (2)1.2 ml PBS (positive control), (3) 1.2 ml rattle-type MSN dispersion in PBS with different concentrations (62.5 μg·ml^−1^, 125 μg·ml^−1^, 250 μg·ml^−1^, 500 μg·ml^−1^, 1 mg·ml^−1^, 2 mg·ml^−1^, 4 mg·ml^−1^). Above mixed dispersion was shaken and kept static for 2 h, and then they were centrifuged for 2 min at 4000 rpm, and the upper solution was taken out to measure the absorption value at 541 nm *via* UV-*vis* absorption spectra. The hemolysis ratio (H_r_) can be obtained according to Lambert-Beer, and the equation is given as follows:
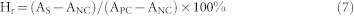
Wherein, A_S_ represents absorbance of sample groups, A_NC_ represents absorbance of negative control, and A_PC_ represents the absorbance of positive control.

### Blood coagulation assay

Firstly, the plasma kindly provided from Shanghai (Red Cross) Blood Center was taken out from frozen plasma. Rattle-type MSN particles were dispersed in PBS solution, and then were diluted into different concentrations (62.5 μg·ml^−1^, 125 μg·ml^−1^, 250 μg·ml^−1^, 500 μg·ml^−1^, 1 mg·ml^−1^, 2 mg·ml^−1^, 4 mg·ml^−1^). The sample dispersion (50 μl) and plasma (450 μl) were mixed with each other, and kept static at room temperature for 5 min, and then the mixture was centrifuged, and their upper solution was collected. According to the instruction of HemosIL™ (Instrumentation Laboratory Company, Lexingtion, MA 02421-325, USA), Calcium Chloride, SynthASil and PT-Fibrinogen HS Plus were added into the corresponding analysis tank of ACLTM200 automic blood coagulation analyzer, and the PT, APTT and FIB was monitored.

### *Intracellular* ultrasound imaging aiming on L929 cell lines

Two vessels of L929 cells were cultured and maintained in high glucose Dulbecco's modified Eagle's medium (DMEM) supplemented with 10% fetal bovine serum at 37°C in a fully humidified atmosphere of 5% CO_2_, and one vessel was labeled with control, and another was labeled with rattle-type MSN. When the number of cells occupied up to 80% of walls, the supernatant in two vessels was removed, and 10 ml (1 mg·ml^−1^) of rattle-type MSN particles dispersion in DMEM with 10% fetal bovine serum was added into the vessel labeled with rattle-type MSN, and 10 ml DMEM with 10% fetal bovine serum was added into the vessel labeled with control. After incubation for another 24 h, in the vessel labeled with rattle-type MSN, the rattle-type MSN particles that were not engulfed were removed *via* PBS wash for 5 times, and then the cells in the two vessels were harvested with 1 ml pancreatin (5%), and then collected *via* 1500 rpm centrifugation for 5 min. Finally, the two groups of collected cells were re-dispersed into 1 ml PBS for ultrasound imaging.

By ICP quantitative test, the Si mass engulfed by L929 cells (the order of 4.0 × 10^8^) is 3 mg that is beneficial for realizing intracellular ultrasound imaging, and the procedures of intracellular ultrasound imaging are the same with that of *in vitro* ultrasound imaging.

### Bio-TEM observation

One vessel of L929 cells was cultured and maintained in high glucose Dulbecco's modified Eagle's medium (DMEM) supplemented with 10% fetal bovine serum at 37°C in a fully humidified atmosphere of 5% CO_2_, and was labeled with rattle-type MSN. When the number of cells occupied up to 80% of walls, the supernatant in two vessels was removed, and 10 ml (0.2 mg·ml^−1^) of rattle-type MSN particles dispersion in DMEM with 10% fetal bovine serum was added into the vessel. After incubation for another 24 h, the rattle-type MSN particles that were not engulfed were removed *via* PBS wash for 5 times, and then the cells were harvested with 1 ml pancreatin (5%), and then collected *via* 1500 rpm centrifugation for 5 min. Afterwards, the collected cells was immbolized with glutaraldehyde fixative (2.5%) for observation.

### Evaluations on *in vivo* blood toxicity

All animal experiments in this study were performed according to protocols approved by the Laboratory Animal Center of Chongqing Medical University and were in accordance with the policies of National Ministry of Health. 12 healthy New Zealand white rabbits, weighing 2.5–3.0 kg were were supplied by Laboratory Animals Center of Chongqing Medical University, and were averaged into 2 groups, and named as control group (n = 6) and experimental group (n = 6), respectively. All rabbits in experimental group were injected with rattle-type MSN particles (3 ml, 8 mg/ml) *via* ear vein, as control group, the rabbits was injected with PBS (3 ml). After 15 days, 12 rabbits were blooded for analysis on COULTER AC Tdiff2 Hematology (BECKMAN COULTER) and the evaluation indicators are the same with those in clinical analysis. After another 15 days, the blood of 6 rabbits in experimental group were collected for analysis.

### *In vivo* solid VX2 tumor imaging in rabbit model

New Zealand white rabbits, weighing 2.5–3.0 kg with or without VX2 liver tumor (4–5 cm^3^) were supplied by Laboratory Animals Center of Chongqing Medical University. VX2 solid tumor was taken out of the legs of VX2 tumor-bearing rabbit, and then was chopped. Moderate chopped VX2 tumor were directly injecting into the livers of healthy rabbits after surgical laparotomy of healthy rabbits, and then sutured the abdominal incision. After 4 weeks, the VX2 solid tumor can be successfully generated in the liver of rabbits for use. Animals were fasted for 24 h before experiments and their abdomen and back were shaved. The experiment was approved ethically and scientifically by the University and complied with Practice for Laboratory Animals in China. After the rabbit was anesthetized and fixed, 2 ml sample dispersion in PBS was injected into tumor *via* the ultrasound-guided percutaneous injection method. Ultrasonic images before and after injecting rattle-type MSN dispersion were recorded under B fundamental imaging mode, tissue harmonic imaging mode and contrast harmonic imaging mode. Under THI mode, the mechanical index (MI) is 0.7 and the frame frequency (FR) is 15 Hz; under BFI mode, the mechanical index (MI) is 0.6 and frame frequency (FR) is 28 Hz; under CHI mode, the mechanical index (MI) is 0.07 and frame frequency (FR) is 9 Hz.

## Author Contributions

K.Z., H.C. and J.S. designed the experiments and wrote the main manuscript text and K.Z. prepared all figures. K.Z., X.G., D.Z., H.Z. and Y.Z. performed the experiments. All authors reviewed the manuscript.

## Supplementary Material

Supplementary Information supplementary information
