# Supplementary material for: Double-scattering/reflection in a Single Nanoparticle for Intensified Ultrasound Imaging
Source: Sci Rep. 2015 Mar 5;5:8766. doi: 10.1038/srep08766 (PMC4350106; doi:10.1038/srep08766)
Supplement: Supplementary Information [file srep08766-s1.doc]

*Supplementary information for*

Double-scattering/reflection in a Single Nanoparticle for Intensified Ultrasound Imaging

By Kun Zhang, Hangrong Chen,* Xiasheng Guo, Dong Zhang, Yuanyi Zheng Hairong Zheng and Jianlin Shi*

Dr. K. Zhang, Prof. J. Shi and Prof. H. Chen

State Key Laboratory of High Performance Ceramics and Superfine Microstructures,

Shanghai Institute of Ceramics, Chinese Academy of Sciences

1295 Ding-Xi Road, Shanghai 200050, P. R. China

Email: [hrchen@mail.sic.ac.cn](mailto:hrchen@mail.sic.ac.cn), [jlshi@sunm.shcnc.ac.cn](mailto:jlshi@sunm.shcnc.ac.cn),

Prof. X. Guo and Prof. D. Zhang

Key Laboratory of Modern Acoustics, MOE, Institute of Acoustics, Department of Physics, Nanjing University, Nanjing 210093, P. R.China

Prof. Y. Zheng

Second Affiliated Hospital of Chongqing Medical University, Chongqing, 400010, P. R. China

Prof. H. Zheng

Shenzhen Institutes of Advanced Technology, Chinese Academy of Sciences, Shenzhen, 518055, P. R.China

Figures and Tables


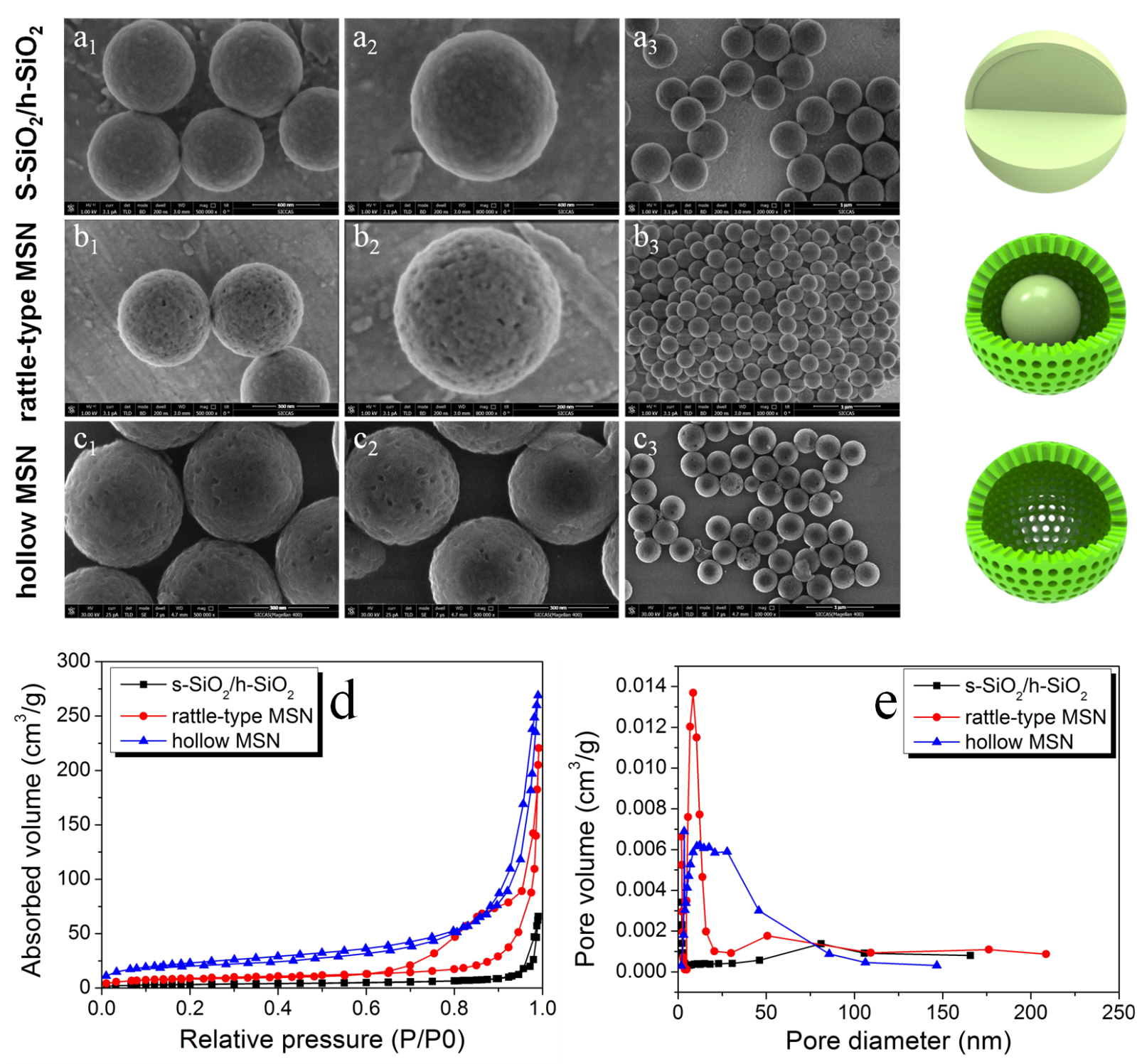


**Fig. S1** Demonstrations of the presence of mesopores in hollow and rattle-type MSNs. (a_1_-a_3_, b_1_-b_3_ and c_1_-c_3_) SEM images of s-SiO2/h-SiO2 (a_1_-a_3_), rattle-type MSN (b_1_-b_3_) and hollow MSN (c_1_-c_3_), respectively; d-e) N_2_ adsorption and desorption isotherms (d) and corresponding pore size distribution (e) of above three structures.

**Table S1.** The actual and theoretical water mass entrapped in the cavities of hollow and rattle structured mesoporous silica nanoparticles with a total particle number of 9.0×10^11^

| **Structure type** | **Particle number** | **Actually entrapped water mass (mg)^§^** | **Theoretically entrapped water mass (mg)^¶^** |
| --- | --- | --- | --- |
| **s-SiO_2_/h-SiO_2_** | 9.0×10^11^ | - | - |
| **hollow MSN** | 9.0×10^11^ | 49.6 | 40.9 |
| **rattle-type MSN** | 9.0×10^11^ | 35.3 | 31.5 |

Notes: § Measured loading mass of water *via* a direct weighing method.

¶ Calculated loading mass of water via hypothesizing the full filling of cavities of rattle-type and hollow MSN particles with neglecting the mesopores in shells, according to the given data of particle diameter, shell thickness and inner core size.


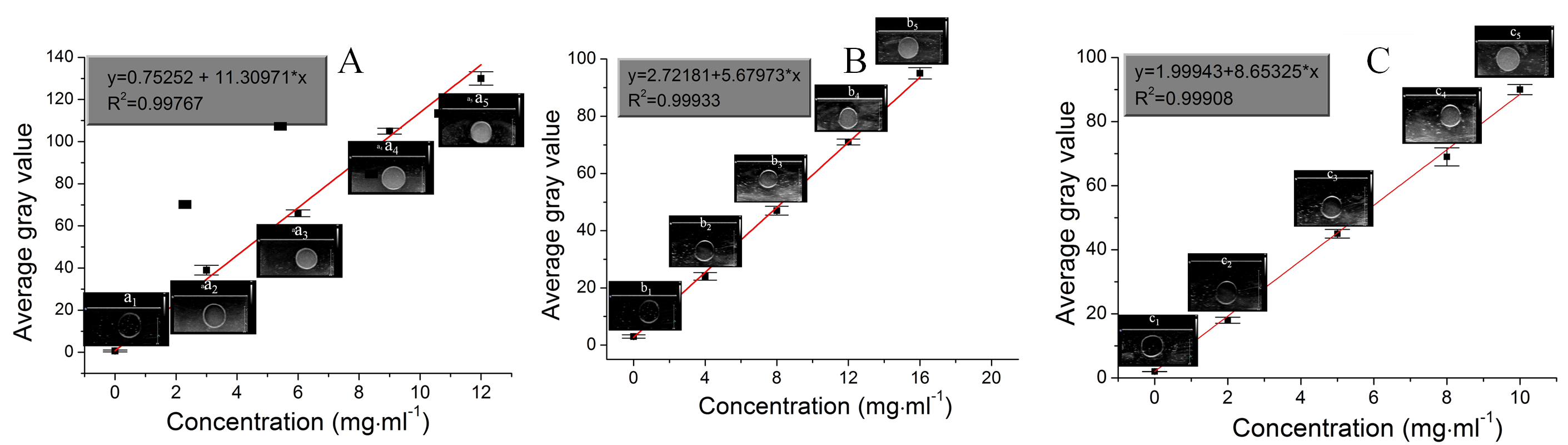


**Fig. S2** The temperature-dependent average gray values of rattle-type MSN, s-SiO_2_/h-SiO_2_ and hollow MSN with different concentrations, respectively, indicating a linear relationship with the particle concentration; and the insets are their corresponding ultrasound images measured under B fundamental imaging mode.


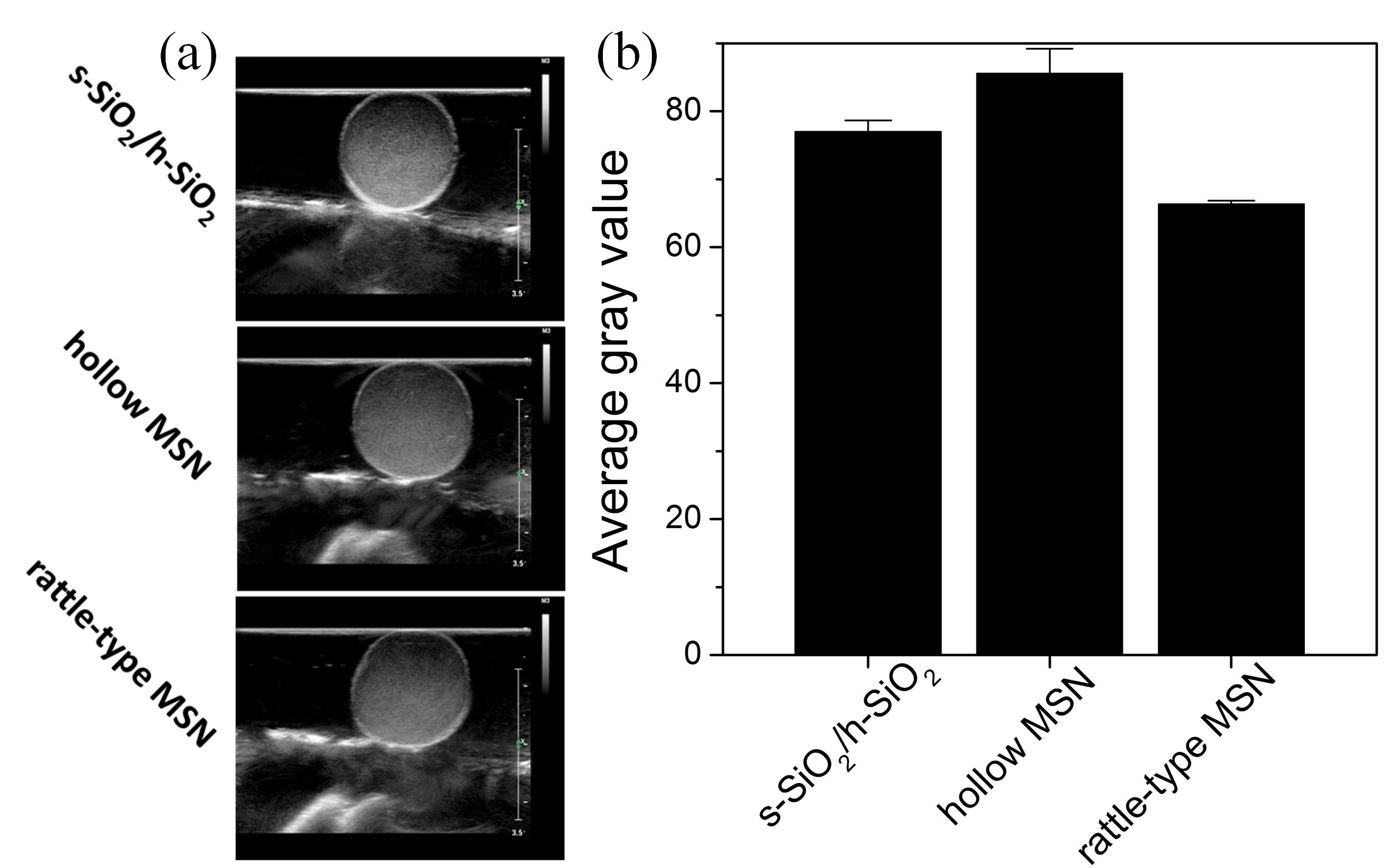


**Fig. S3** Ultrasound imaging results of three different structures with the same number of backscattering interfaces *via* reducing the particle concentration of rattle-type MSN to the half of solid or hollow MSN. a) Ultrasonic images of the three different structured particles under B fundamental imaging mode; b) Their corresponding average gray values.


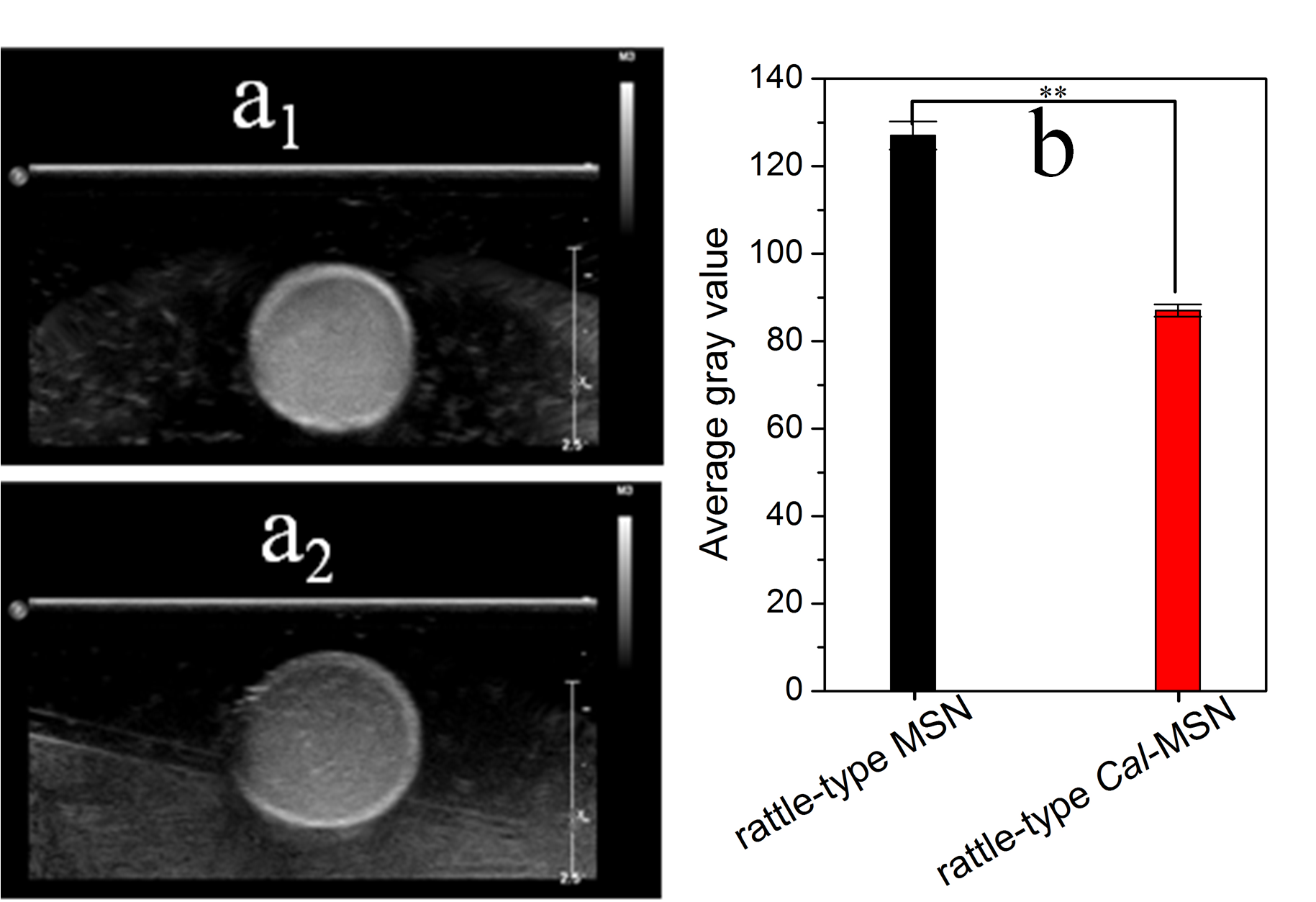


**Fig. S4** a_1_-a_2_) Ultrasonic images of rattle-type MSN (a_1_) and its corresponding calcined one, rattle-type *cal*-MSN deriving from the calcinations of rattle-type MSN under B fundamental imaging mode (a_2_); b) Measured average gray values of rattle-type MSN and rattle-type *cal*-MSN. Note: ** represents significant difference in average gray value by comparing rattle-type MSN and rattle-type *cal*-MSN at P ≤ 0.01


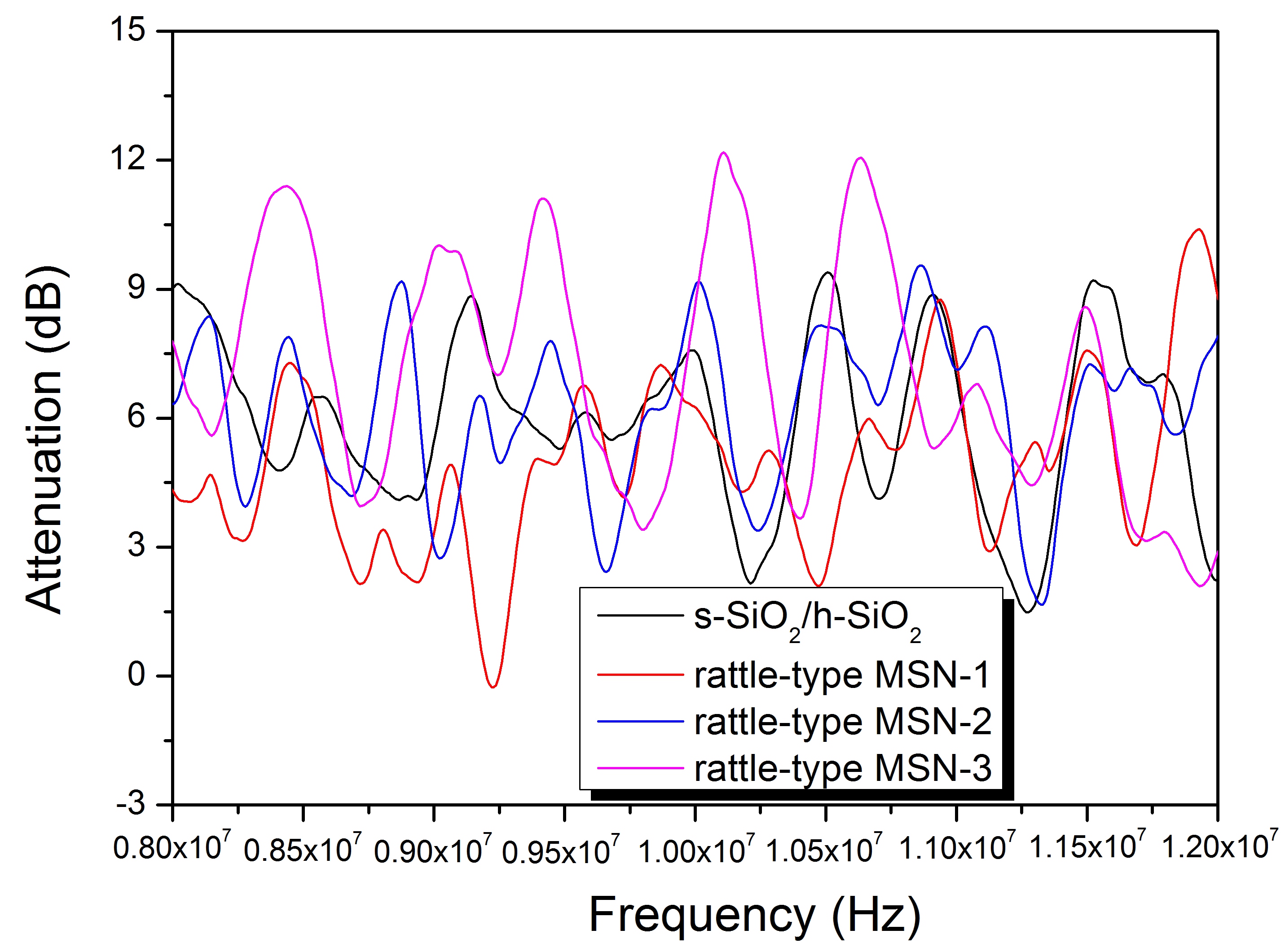


**Fig. S5** Measurement results of reflection contributions: attenuation-frequency characteristic of sound in suspensions containing s-SiO_2_/h-SiO_2_, rattle-type MSN-1, rattle-type MSN-2 and rattle-type MSN-3 (the same particle concentration of 2.65×10^7^ per ml), respectively; Notes：Employing the same plane transducer (center frequency at 10 MHz) with a 3-dB bandwidth to emit and receive signals.


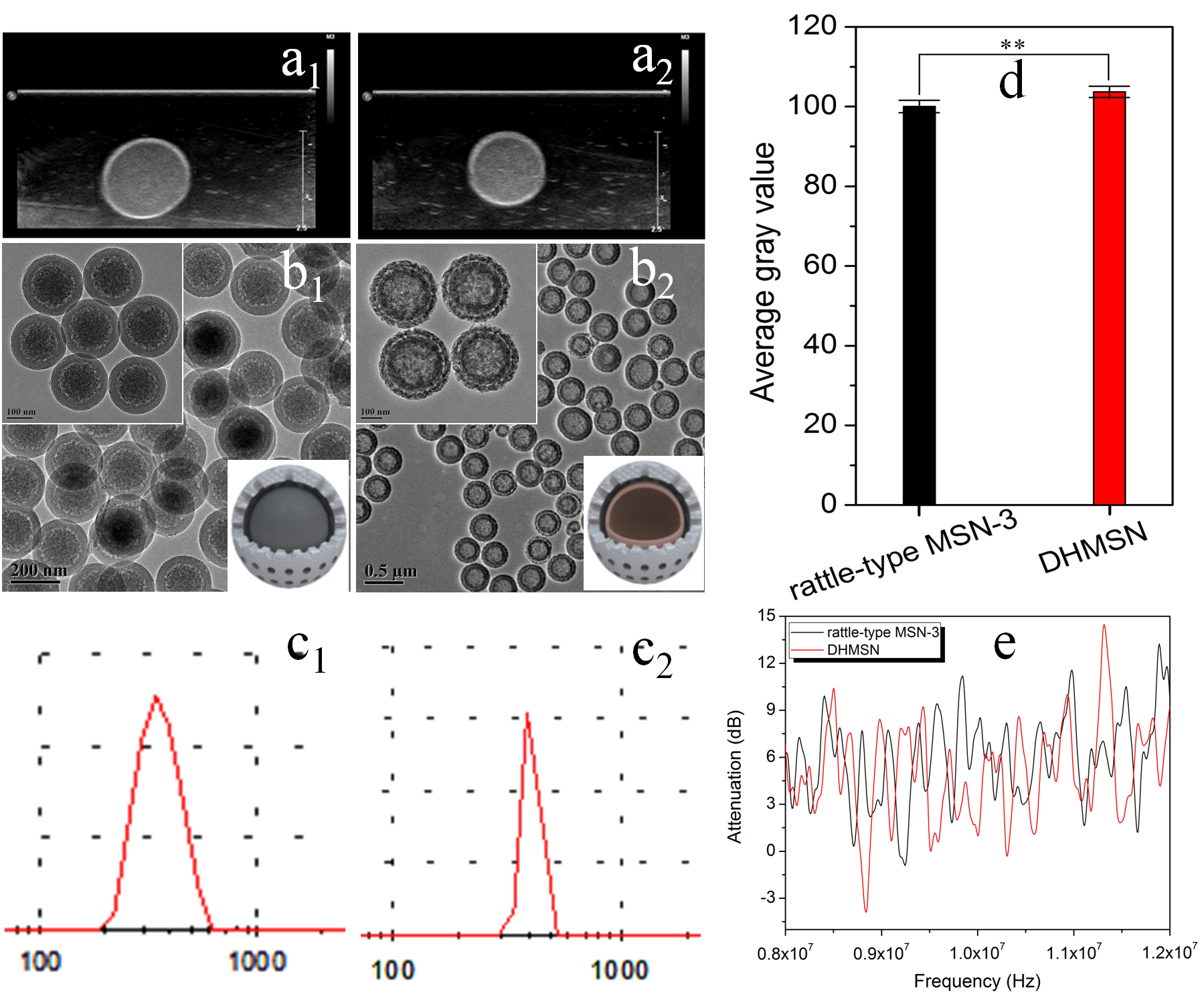


**Fig. S6** a_1_-a_2_) Ultrasound images of rattle-type MSN-3 and DHMSNs under B fundamental imaging mode with an excitation frequency centering at 10 MHz; b_1_-b_2_) TEM images of rattle-type MSN-3 and DHMSNs, respectively, and insets are their respective 3-D model; c_1_-c_2_) DLS values of rattle-type MSN-3 and DHMSNs; and d) Measured average gray values of rattle-type MSN-3 and DHMSNs. Both share the same number of interfaces and the same radius of both shell and inner core (1^st^ and 2^nd^), but the structure of their inner cores are different (e.g. for DHMSN, the 2^nd^ interface is hollow silica sphere, while for rattle-type MSN-3, the 2^nd^ interface is solid silica sphere); e) Attenuation-frequency characteristic of sound in suspensions containing rattle-type MSN-3 and DHMSN both of which share the same particle concentration of 2.65×10^7^ per ml, respectively. Note: ** represents significant difference in average gray value by comparing rattle-type MSN-3 with DHMSN at P ≤ 0.01.


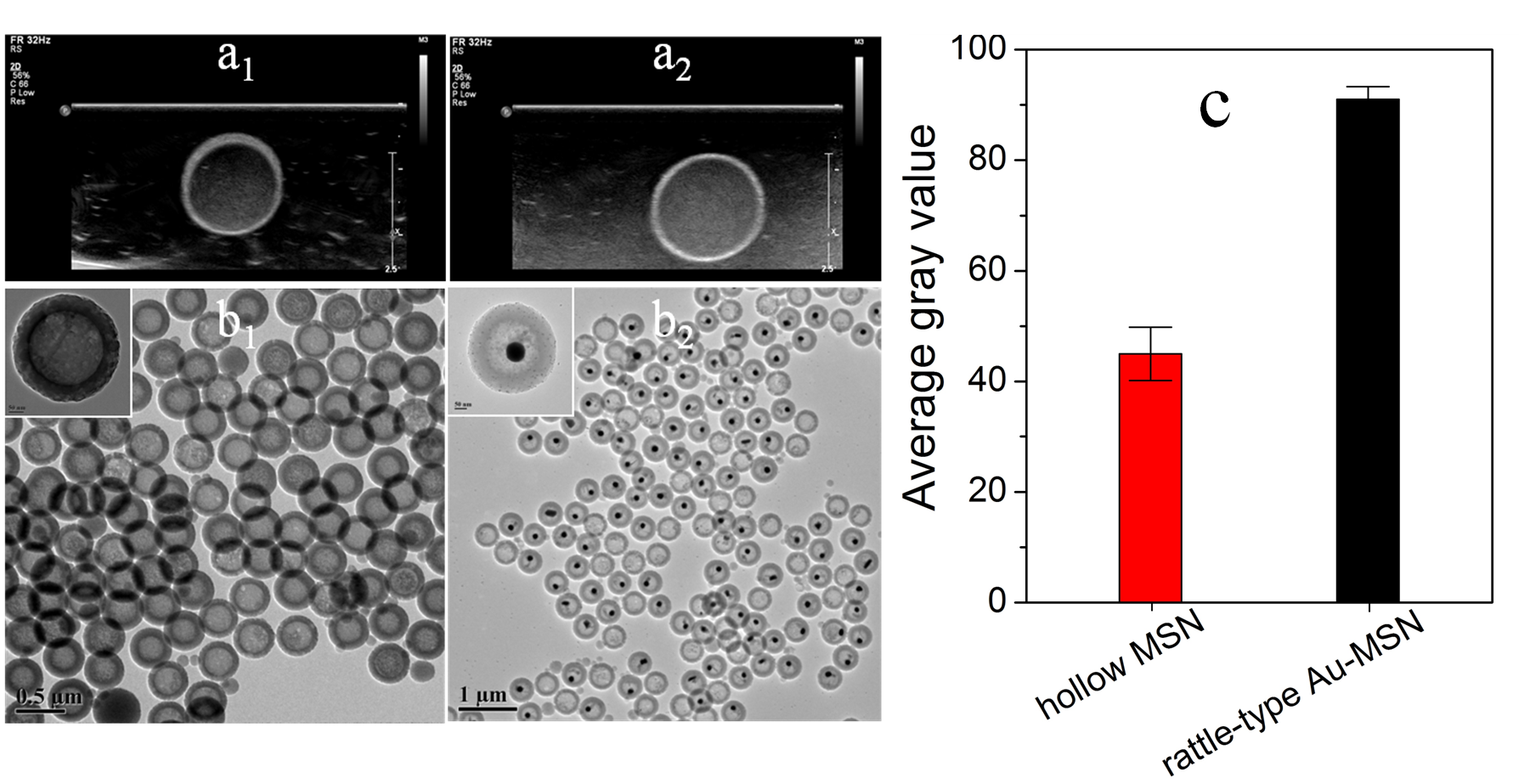


**Fig. S7** a_1_-a_2_) Ultrasonic images of hollow MSN (a_1_) and rattle-type Au-MSN (a_2_) B fundamental imaging mode; b_1_-b_2_) TEM images of hollow MSN (b_1_) and rattle-type Au-MSN (b_2_); c) Corresponding average gray values of hollow MSN and rattle-type Au-MSN originated from a_1_ and a_2_, respectively.


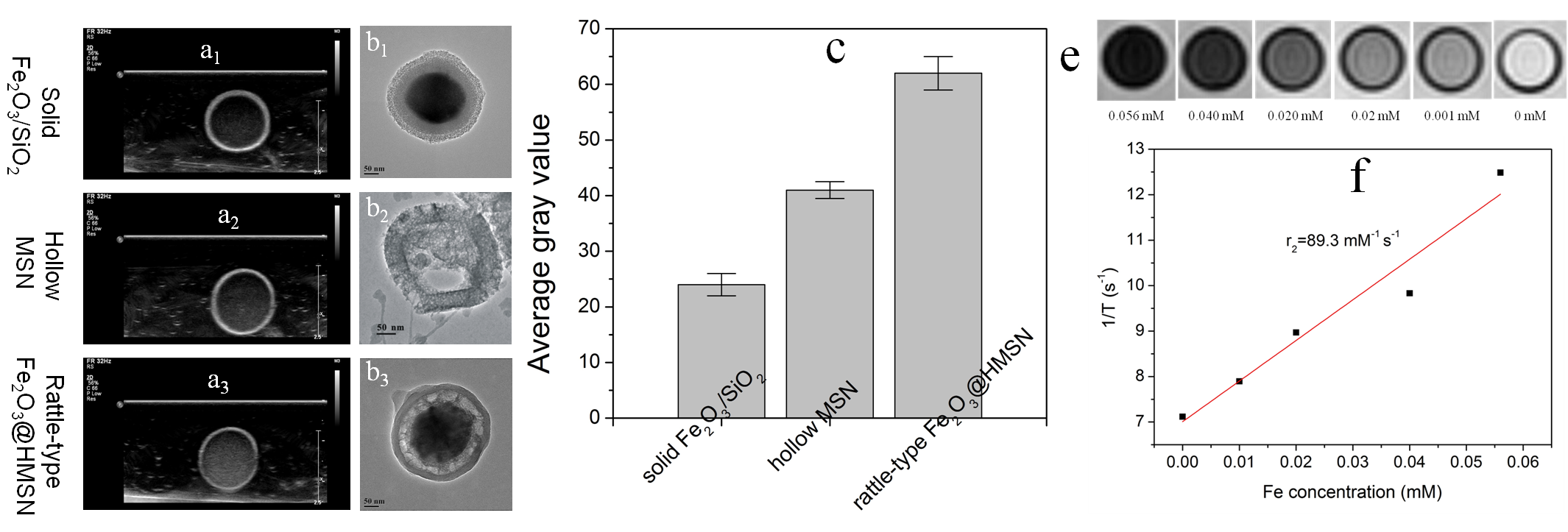


**Fig. S8** a_1_-a_3_) Ultrasonic images of employing solid Fe_2_O_3_/SiO_2_ (a_1_), hollow MSN (a_2_) and rattle-type Fe_2_O_3_@HMSN (a_3_) as UCAs under B fundamental imaging mode; b_1_-b_3_) TEM images of solid Fe_2_O_3_/SiO_2_ (b_1_), hollow MSN (b_2_) and rattle-type Fe_2_O_3_@HMSN (b_3_); c) Corresponding average gray values of employing solid Fe_2_O_3_/SiO_2_, hollow MSN and rattle-type Fe_2_O_3_@HMSN as UCAs; e,f) T2-weighted MR images of different Fe molar concentrations (e) and calculated r_2_ value (f).


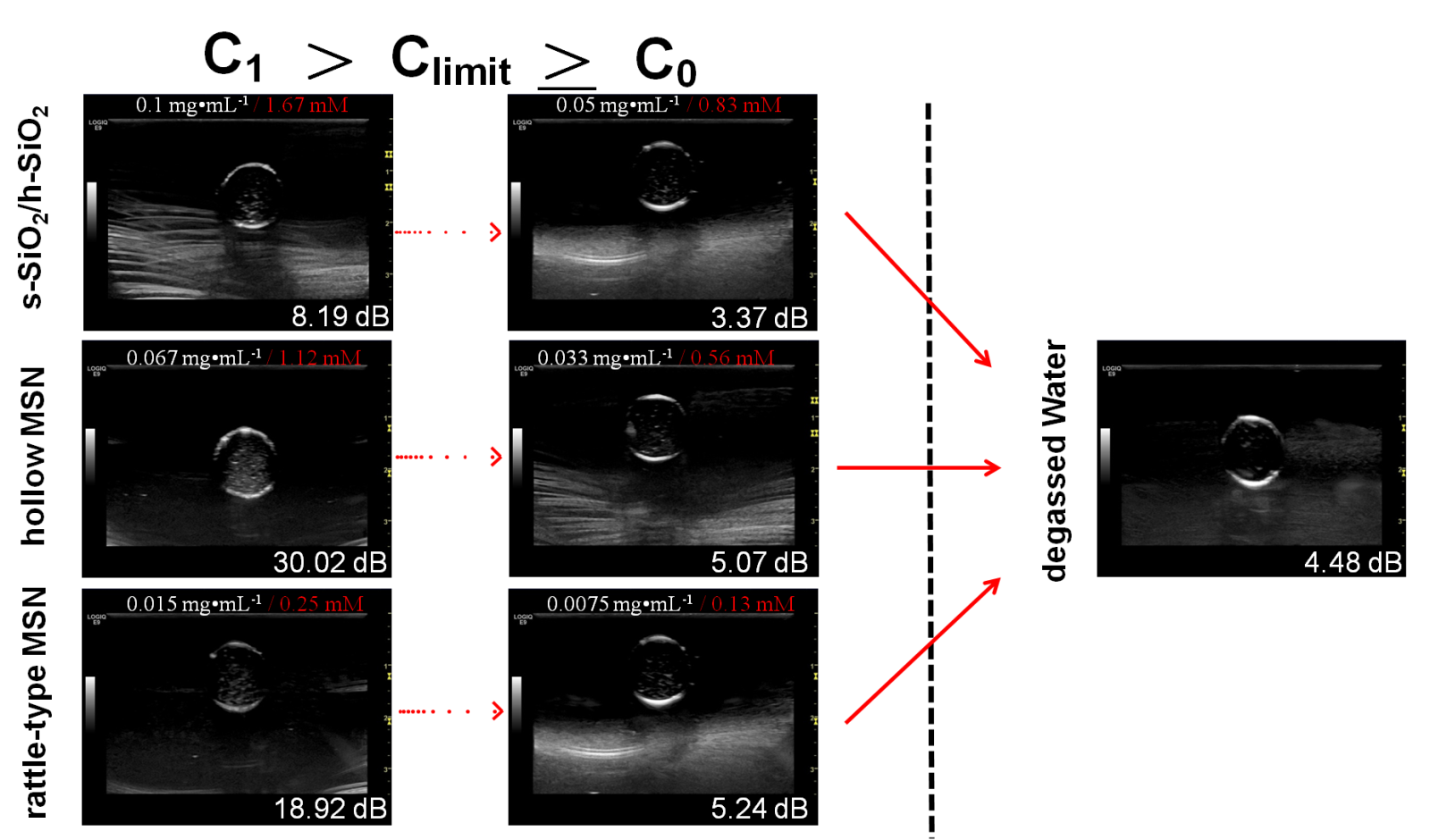


**Fig. S9** Measurements of ultrasonic detection limits of s-SiO_2_/h-SiO_2_, hollow MSN and rattle-type MSN *via* gradually dilution method under B fundamental imaging mode, and c_1_ and c_0_ represent the penult and tailender mass concentrations (molar concentrations). Notes: at C_0_, their signals and contrasts are close to that of degassed water, while at C_1_, their signals are stronger than that of degassed water, which determines that the C_limit_ should be in between C_1_ and C_0_.


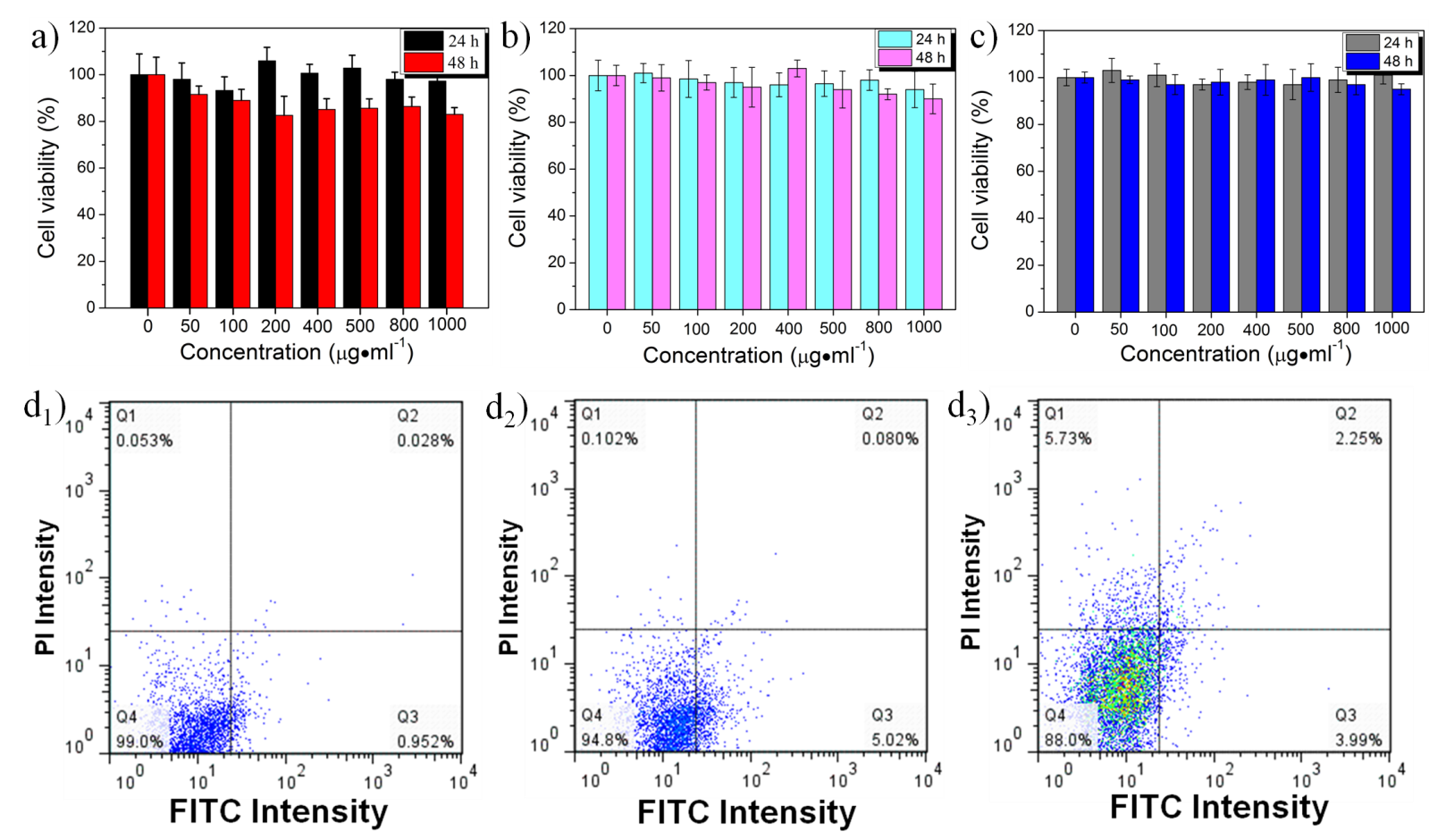


**Fig. S10** a-c) Cell viabilities of L-929, BCECs and Hela cell lines treated with rattle-type MSN nanoparticles of different mass concentrations *via* MTT method, d_1_-d_3_) Cytotoxicity of L929 treated with rattle-type MSN particles of different mass concentrations, 0 (d_1_), 0.5 mg∙ml^-1^ (d_2_) and 1 mg∙ml^-1^ (d_3_) *via* flow cytometry.


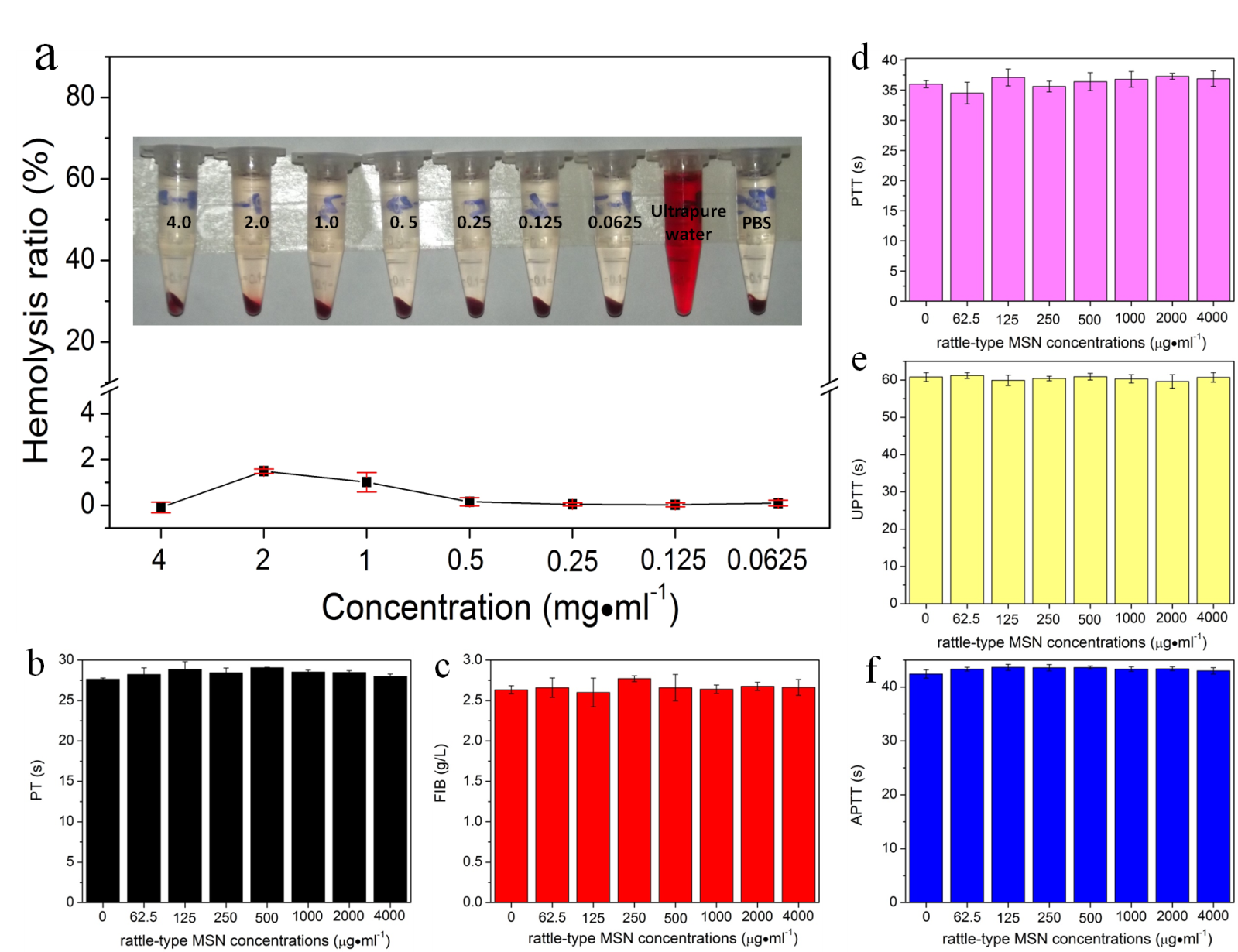


**Fig. S11** a) Hemolysis assay for rattle-type MSN particles at different mass concentrations; b-f) Blood coagulation assay for rattle-type MSN particles, and measured PT (b), FIB (c), PTT (d), UPTT (e) and APTT (f) values of blood plasma after the exposure to rattle-type MSN of different mass concentrations. Notes: PT: prothrombin time; FIB: fibrinogen; APTT: activated partial thromboplastin time; PTT: partial thromboplastin time, UPTT:. unactivated partial thromboplastin time.


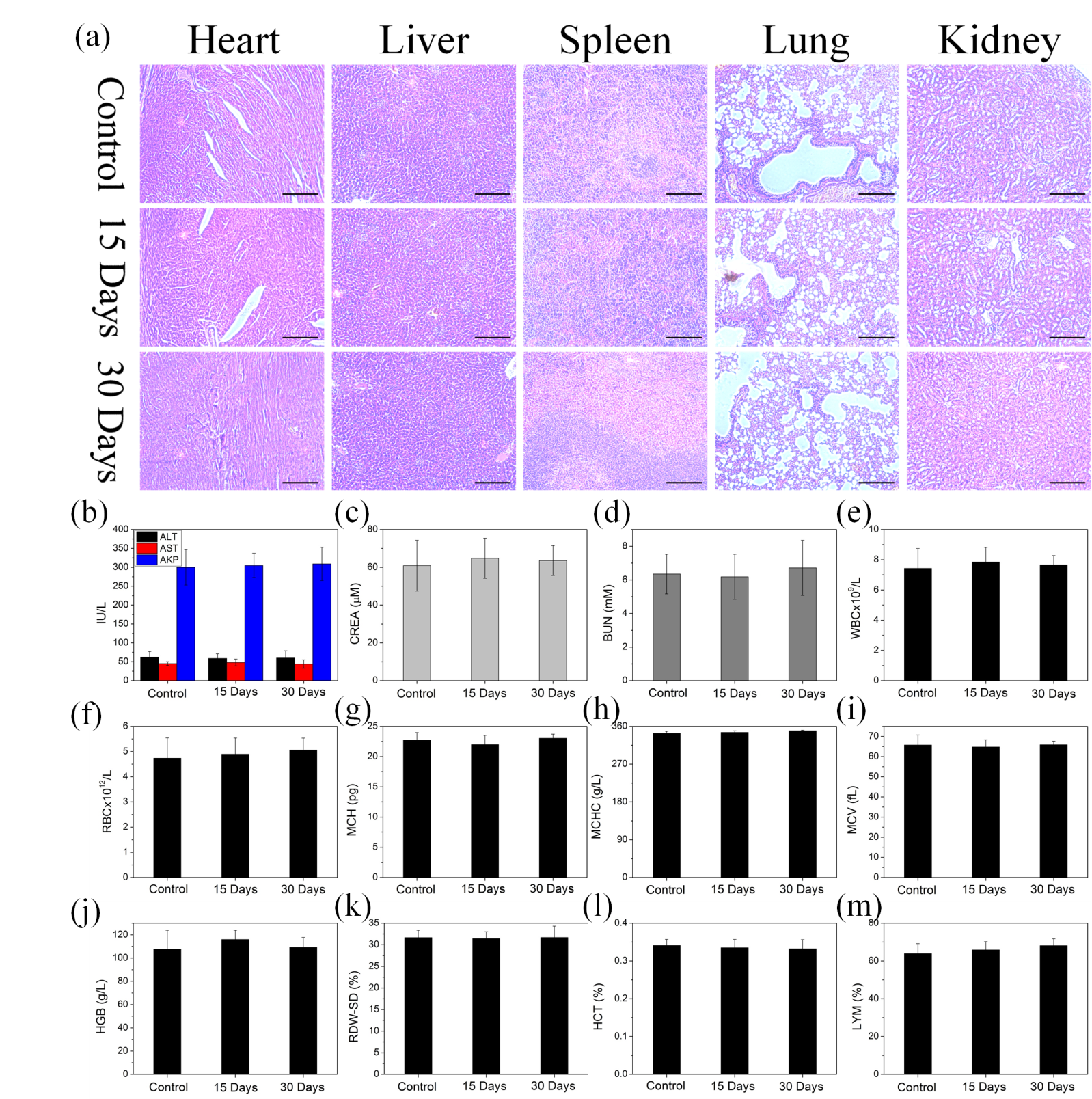


**Fig. S12** *In vivo* toxicity investigation results of rattle-type MSNs. (a) H&E-stained tissues from main organs of New Zealand white rabbits to monitor the histological changes after the intravenous injection of rattle-type MSN (8 mg /kg). Scale bar: 200 μm; b-m) Blood biochemistry data obtained from New Zealand white rabbits after the intravenous injection of rattle-type MSN (n = 3, dose = 8 mg /kg) at various time points or receiving no injection as control.


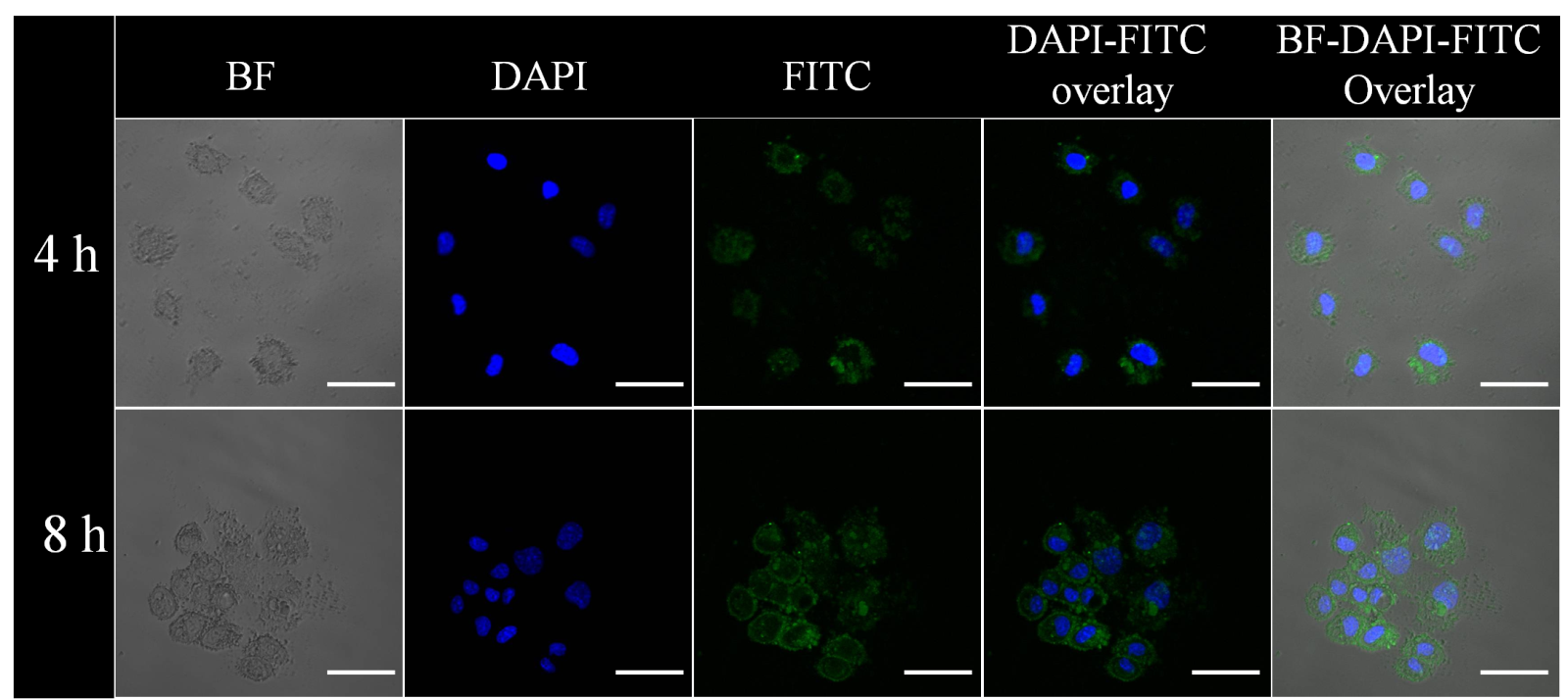


**Fig. S13** Internalization test of rattle-type MSN nanoparticles with a concentration of 100 ug∙ml^-1^ by L929 cells in different time intervals (4 h and 8 h). BF represents bright field, FITC represents FITC-labeled rattle-type MSN, and the scale bar is 50 μm.


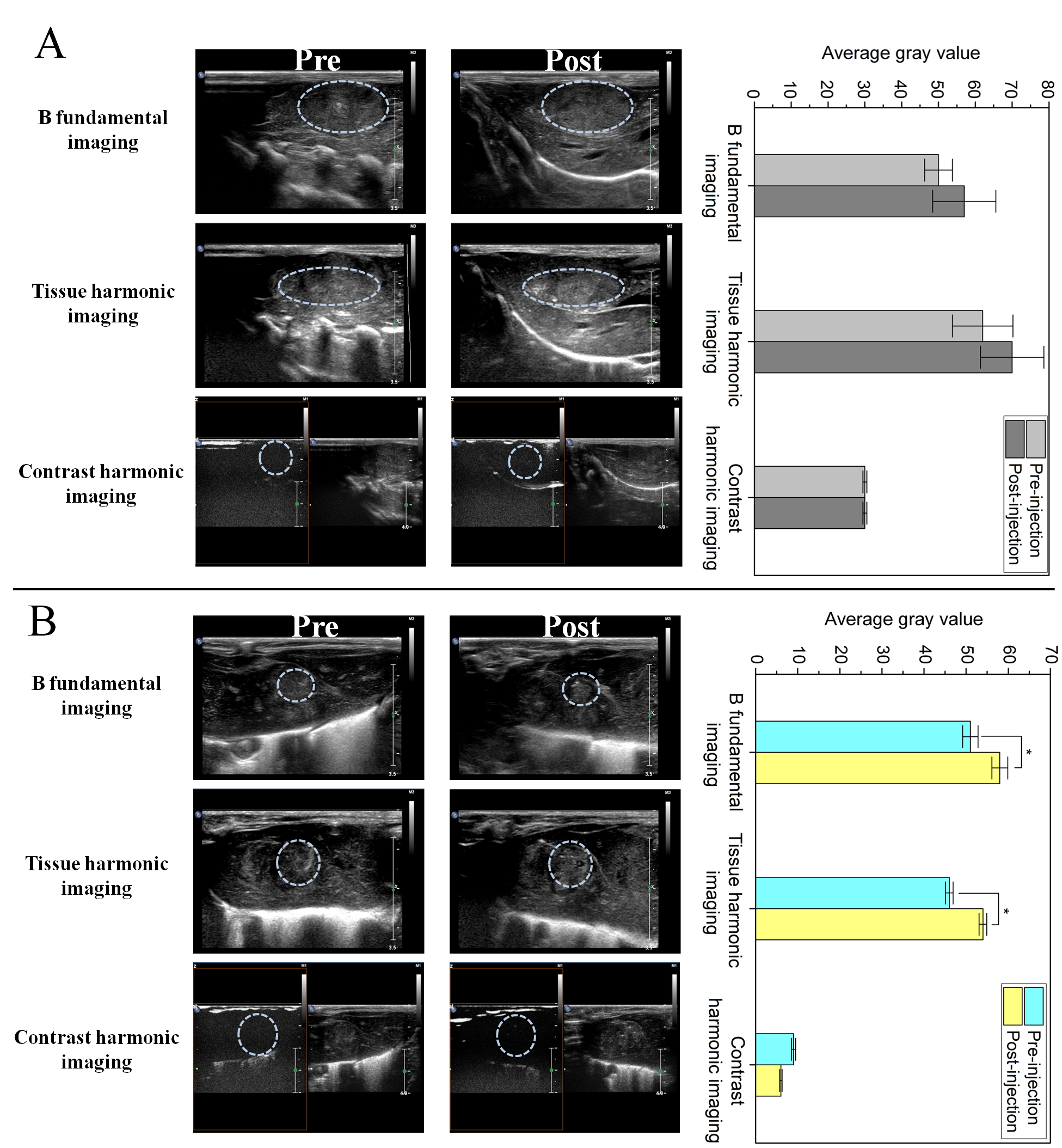


**Fig. S14** a-b) *In vivo* ultrasound images and corresponding average gray values of VX2 liver tumor in the rabbit model under B fundamental imaging, tissue harmonic and contrast harmonic imaging before and after injecting s-SiO_2_/h-SiO_2_ (A) and hollow MSN (B) of a particle concentration of 10^8^ orders (circled by dotted ellipse). * represents significant difference in average gray values by comparing before injecting rattle-type MSN with after injecting hollow MSN at P ≤ 0.05.


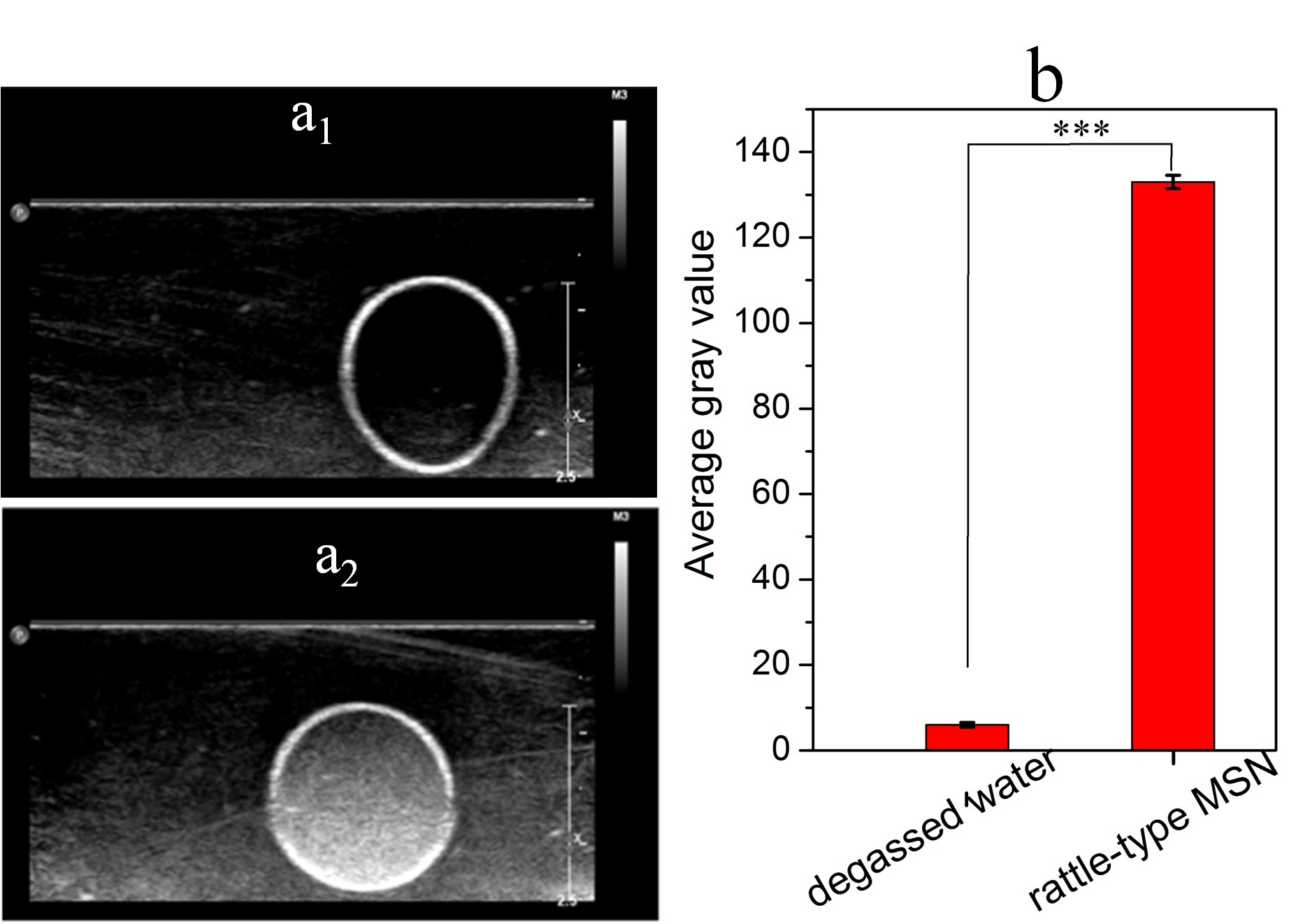


**Fig. S15** a_1_-a_2_) Ultrasound images of PBS and rattle-type MSN under tissue harmonic imaging mode; b) the measured average gray values of PBS and rattle-type MSN under tissue harmonic imaging mode. Note: *** represents significant difference in average gray value by comparing degassed water with rattle-type MSN at P ≤ 0.001.
